# Understanding the diversity and roles of the canine gut microbiome

**DOI:** 10.1186/s40104-025-01235-4

**Published:** 2025-07-05

**Authors:** Haram Kim, Yeongjae Chae, Jin Ho Cho, Minho Song, Jinok Kwak, Hyunok Doo, Yejin Choi, Juyoun Kang, Hyunjin Yang, Suyoung Lee, Gi Beom Keum, Suphot Wattanaphansak, Sheena Kim, Hyeun Bum Kim

**Affiliations:** 1 Department of Animal Biotechnology, Dankook University, Cheonan, 31116 South Korea; 2 Division of Food and Animal Science, Chungbuk National University, Cheongju, 28644 South Korea; 3 Division of Animal and Dairy Science, Chungnam National University, Daejeon, 35015 South Korea; 4 Department of Veterinary Medicine, Faculty of Veterinary Science, Chulalongkorn University, Bangkok, 10330 Thailand

**Keywords:** Canine, Gut health, Gut microbiome, Microbiome balance

## Abstract

The canine gut microbiome plays a vital role in overall health and well-being by regulating various physiological functions, including digestion, immune responses, energy metabolism, and even behavior and temperament. As such, a comprehensive understanding of the diversity and functional roles of the canine gut microbiome is crucial for maintaining optimal health and well-being. In healthy dogs, the gut microbiome typically consists of a diverse array of bacterial phyla, including Firmicutes, Bacteroidetes, Actinobacteria, Fusobacteria, and Proteobacteria. These microbial communities form a complex ecosystem that interacts with the host to support canine health and homeostasis. A well-balanced microbiome, known as eubiosis, represents an optimized microbial composition that enhances host health and metabolic functions. Eubiosis is shaped by interactions between host physiology and environmental factors. However, dysbiosis, a disruption of eubiosis, can contribute to various health issues, such as weight fluctuations, metabolic disorders, and behavioral changes. Maintaining eubiosis in the canine gut microbiome requires customized management strategies that consider both physiological traits and environmental influences. In this review, we explored the structure and function of the canine gut microbiome, with particular emphasis on its role in health and the key factors that influence and support its maintenance.

## Introduction

Canines have been domesticated and have coexisted with humans for thousands of years, with most owners considering them family members or cherished companions. Moreover, the number of companion animals in modern society is increasing, with canines becoming significant human companions [1]. Given this trend, there is growing interest in the health and well-being of companion canines [2].

The canine gut microbiome plays a crucial role in maintaining overall health and well-being by influencing various physiological functions, including digestion, immune regulation, energy metabolism, and even behavior and temperament. The gut microbiome of healthy companion canines typically comprises diverse bacterial phyla, such as Firmicutes, Bacteroidetes, Actinobacteria, Fusobacteria, and Proteobacteria [3–6]. These microbial communities form a complex and dynamic ecosystem that closely interacts with the host to support canine health and homeostasis.

A well-balanced microbiome, known as eubiosis, represents an optimized microbial composition that promotes host health and metabolic functions. Eubiosis is shaped by interactions between host physiology and environmental conditions. Biological factors such as age and stress levels critically influence microbial diversity and composition [7]. For example, a recent study analyzing the canine gut microbiome at 15-day intervals revealed that microbial composition can shift even within short periods [8]. This study highlights the microbiota’s sensitivity to age and growth stages, which can have both direct and indirect effects on canine health and welfare. Furthermore, gut microbiota composition can be significantly altered by other factors, such as antibiotic use. Dysbiosis, an imbalance in the gut microbiome, can lead to health issues such as weight fluctuations, metabolic disorders, and behavioral changes [9–14]. In addition, the composition and activity of the gut microbiome are closely linked to disease prevention [15, 16]. A stable and well-developed gut microbiota contributes to host defense by preventing colonization by harmful microorganisms. It works in concert with the host’s defense mechanisms, particularly the gut-associated immune system, to resist pathogen invasion [17]. However, when eubiosis is disrupted, dysbiosis may result in an overgrowth of harmful bacteria and a decline in beneficial microbes. This imbalance can trigger immune dysregulation and negatively affect overall well-being [7, 18].

To objectively assess canine gut microbial imbalance, the canine microbiota dysbiosis index (CMDI) was developed [19, 20]. The CMDI is calculated using quantitative PCR analysis of seven major bacterial taxa, comprising both beneficial and potentially harmful bacteria, associated with dysbiosis in canine feces. This allows for the quantification of alterations in the intestinal microbiota. This index is widely used in both clinical and research settings to diagnose intestinal microbiota imbalance and to objectively monitor changes over time [21, 22]. However, beyond such assessment tools, targeted strategies are required to effectively maintain and restore intestinal balance. These strategies should be tailored through a customized management approach that accounts for both physiological characteristics and environmental influences. Strategies to maintain a balanced gut microbiome include providing a diet optimized for the canine’s growth stage, ensuring appropriate levels of protein, fiber, and carbohydrates [23–25]. The incorporation of probiotics and prebiotics into the diet is also essential, as they promote the growth of beneficial bacteria and help sustain eubiosis within the gastrointestinal tract [26, 27]. Additionally, restoring beneficial microbial populations following antibiotic use, minimizing exposure to stressors, and ensuring a stable external environment are effective methods for maintaining microbiome balance [28–30].

In conclusion, a comprehensive understanding of the diversity and function of the canine gut microbiome, as well as the intrinsic and extrinsic factors that influence and support its maintenance, is crucial for improving canine health and well-being. In this review, we explored the structure and function of the canine gut microbiome, with particular emphasis on its role in health and the key factors that influence and support its maintenance.

## General composition of the canine gut microbiome and its related functions

The canine gut microbiome is composed of a diverse array of bacterial strains. In healthy canines, the dominant bacterial phyla including Firmicutes, Bacteroidetes, Actinobacteria, Fusobacteria, and Proteobacteria contribute to a complex and dynamic gut ecosystem [3–6]. However, the composition of the gut microbiome can vary depending on the location within the gastrointestinal tract (GIT), as each section presents unique environmental conditions, such as oxygen tension, nutrient availability, and pH levels. These factors lead to variations in microbial distribution [31, 32].

### Microbial composition in different sections of the GIT

**Fig. 1:**
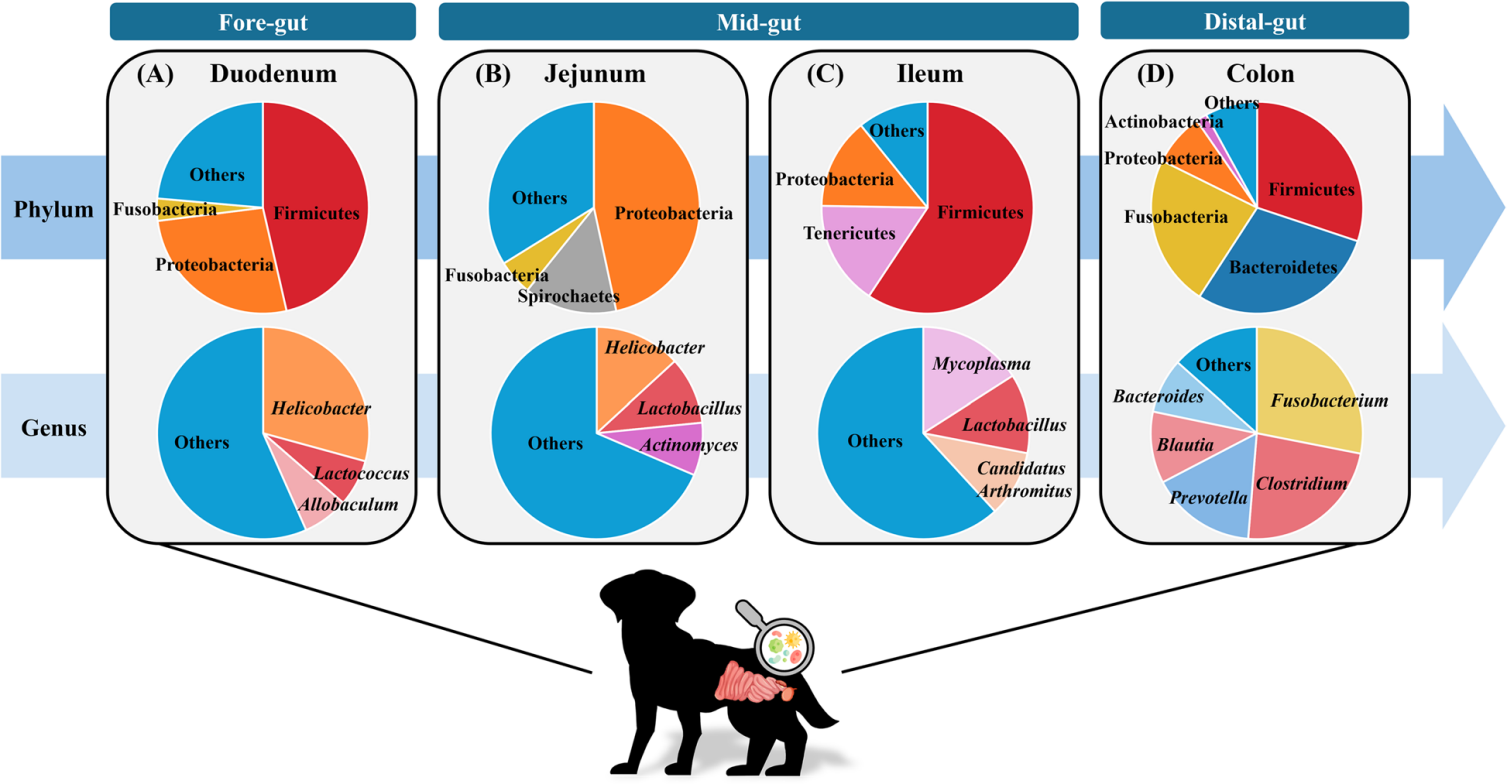
Composition of the canine gut microbiome. (**A)** Microbial compositions at phylum and genus levels in the duodenum. (**B**) Microbial compositions at phylum and genus levels in the jejunum. (**C**) Microbial compositions at phylum and genus levels in the ileum. (**D**) Microbial compositions at phylum and genus levels in the colon. (The figure was created using freely available images from Freepik: https://www.freepik.com/)

In the duodenum, the phyla Firmicutes (46.4%), Proteobacteria (26.6%), and Fusobacteria (3.3%–3.6%) are present in relatively high proportions [5]. At the genus level, *Helicobacter* (29.27%), *Lactococcus* (7.12%), and *Allobaculum* (6.96%) are predominant [33–36] (Fig. 1A). In the jejunum, the dominant phyla include Proteobacteria (46.7%), Spirochaetes (14.2%), and Fusobacteria (5.4%) [37], with *Helicobacter* (13.17%), *Lactobacillus* (10.24%), and *Actinomyces* (8.05%) identified as the dominant genera [33, 36] (Fig. 1B). In the ileum, Firmicutes (59.32%), Tenericutes (15.96%), and Proteobacteria (13.9%) are the predominant phyla, while *Mycoplasma* (15.95%), *Lactobacillus* (12.08%), and *Candidatus Arthromitus* (10.16%) are the most abundant genera [33] (Fig. 1C).

Fecal 16S rRNA gene analysis has shown that the canine distal gut microbiome is primarily composed of Firmicutes, Bacteroidetes, Actinobacteria, Fusobacteria, and Proteobacteria [8, 38] (Fig. 1D). Among these, Firmicutes is the most abundant, typically comprising 15.81%–44.8% of the total gut microbiome [38–40], with some studies reporting levels as high as 75% [8]. Bacteroidetes is the second most dominant phylum, accounting for approximately 30%–36.75% of the total gut microbiome. This group includes beneficial genera such as *Bacteroides* and *Prevotella*, which play essential roles in carbohydrate metabolism and gut health [8, 38, 39]. Actinobacteria makes up 0.33%–3.4% of the gut microbiome and includes *Bifidobacterium*, a genus known for supporting digestion and enhancing immune function [8, 38].

### Roles of major bacterial phyla and key genera in the canine gut microbiome

Firmicutes, Bacteroidetes, and Actinobacteria include beneficial bacteria that play critical roles in digestion and immune function. These bacteria are involved in the breakdown of dietary fiber and carbohydrates, leading to the production of short-chain fatty acids (SCFAs). SCFAs contribute to host energy metabolism, lower pH to inhibit pathogens, and protect the intestinal mucosa to enhance immune responses [3, 41]. Fusobacteria, though less common in other animal species, is frequently found in the canine gut, with reported abundance levels ranging from 8.64% to 39.17% [8, 39, 42]. Although *Fusobacterium* species are part of the normal microbiota in dogs, certain species, such as *Fusobacterium nucleatum*, can exacerbate gut inflammation and contribute to intestinal diseases [43, 44]. Proteobacteria, which comprise 5%–15.26% of the canine gut microbiome, includes pathogenic bacteria such as *Escherichia coli* (*E. coli*), which produce toxins that promote inflammation [8, 36, 42, 45]. While Fusobacteria and Proteobacteria are less abundant than other phyla, they play crucial roles in maintaining gut balance. However, their overgrowth is often indicative of dysbiosis and has been linked to gut-related disorders, such as inflammatory bowel disease (IBD).

At the genus level, the canine distal gut microbiome is dominated by *Fusobacterium*, *Prevotella*, *Blautia*, *Bacteroides*, and *Clostridium,* each of which plays a distinct functional role in gut health [40, 46–52] (Fig. 1D and Table 1).

**Table 1:** Functional roles of key gut bacteria in canines

Key bacteria	Functional roles	References
**Firmicutes**	SCFAs production, immune modulation, energy metabolism	[3, 39, 41, 53, 54]
* Lactobacillus*	SCFAs (lactate) production, pathogen inhibition	[15, 26, 41]
* Allobaculum*	SCFAs (acetate) production, body weight regulation	[33–35]
* Blautia*	SCFAs (acetate) production, gut pH regulation, pathogen inhibition	[55]
* Clostridium*	SCFAs (butyrate) production, gut barrier protection, immune modulation	[56]
* Faecalibacterium*	SCFAs (butyrate) production, anti-inflammatory responses	[39]
* Enterococcus*	SCFAs (acetate) production, anti-inflammatory responses, immune modulation	[15]
**Bacteroidetes**	SCFAs production, immune modulation, energy metabolism	[3, 38–41]
* Prevotella*	SCFAs (acetate) production, carbohydrate and fiber degradation	[15, 47, 49, 50, 57]
* Bacteroides*	SCFAs (acetate) production, fiber and polysaccharide degradation	[58]
**Actinobacteria**	SCFAs production, immune modulation, energy metabolism	[3, 41]
* Bifidobacterium*	SCFAs (lactate, acetate) production, pathogen inhibition, gut barrier protection	[59]
**Fusobacteria**	SCFAs (butyrate) production, inhibition of colorectal cancer	[43, 44]
* Fusobacterium*	Amino acid fermentation, protein metabolism, inflammation modulation	[60]
**Proteobacteria**	Energy metabolism	[8, 15, 36, 42]
* Helicobacter*	Urea degradation, gut pH regulation	[36]

*Fusobacterium* belongs to the Fusobacteria phylum and plays a role in protein degradation and amino acid fermentation, producing butyrate, which supports protein metabolism and inflammation regulation [60]. It is typically found at levels ranging from 7% to 14.3%, with some studies reporting levels as high as 30.52%–49.20%, particularly in response to protein-rich diets and environmental factors [40, 46, 49, 50, 52]. *Prevotella* belongs to the Bacteroidetes phylum and is involved in carbohydrate and fiber degradation, contributing to energy metabolism through SCFAs production [61]. Its abundance in the canine gut typically ranges from 4% to 9.6%, though some studies have reported levels as high as 28.10% due to dietary and environmental variations [47, 49, 50, 62]. *Blautia* belongs to the Firmicutes phylum and is known for producing SCFAs, particularly acetate, which helps regulate gut pH, inhibits pathogen growth, and serves as an energy source for intestinal epithelial cells [55]. It is typically detected in the canine gut at levels ranging from 6.87% to 15% [48–50]. *Bacteroides,* a genus within the Bacteroidetes phylum, plays a key role in fiber and polysaccharide degradation, producing SCFAs that provide energy and contribute to immune regulation [58]. Its abundance in the canine gut ranges from 2% to 14.63% [47, 49, 50, 52, 63]. *Clostridium* belongs to the Firmicutes phylum and is known to produce butyrate which helps protect the intestinal lining and regulate immune responses [56]. It is typically found in the canine gut at levels between 12.69% and 33.7% [49–52, 64]. However, certain pathogenic species, such as *Clostridium perfringens* (*C. perfringens*), can produce toxins that can lead to acute diarrhea and gut inflammation [51, 56].

Overall, the canine gut microbiome consists of a diverse and dynamic microbial community that varies across different sections of the GIT due to unique environmental conditions. While phyla such as Firmicutes, Bacteroidetes*,* and Actinobacteria play essential roles in digestion, immune regulation, and metabolic function, the presence of Fusobacteria and Proteobacteria must be carefully regulated to maintain gut health. Understanding the distribution and functional roles of microbial communities in the canine gut is crucial for developing targeted strategies to maintain gut microbiome balance and support overall health and canine well-being.

## Factors influencing the balance of the gut microbiome in companion canines

**Fig. 2:**
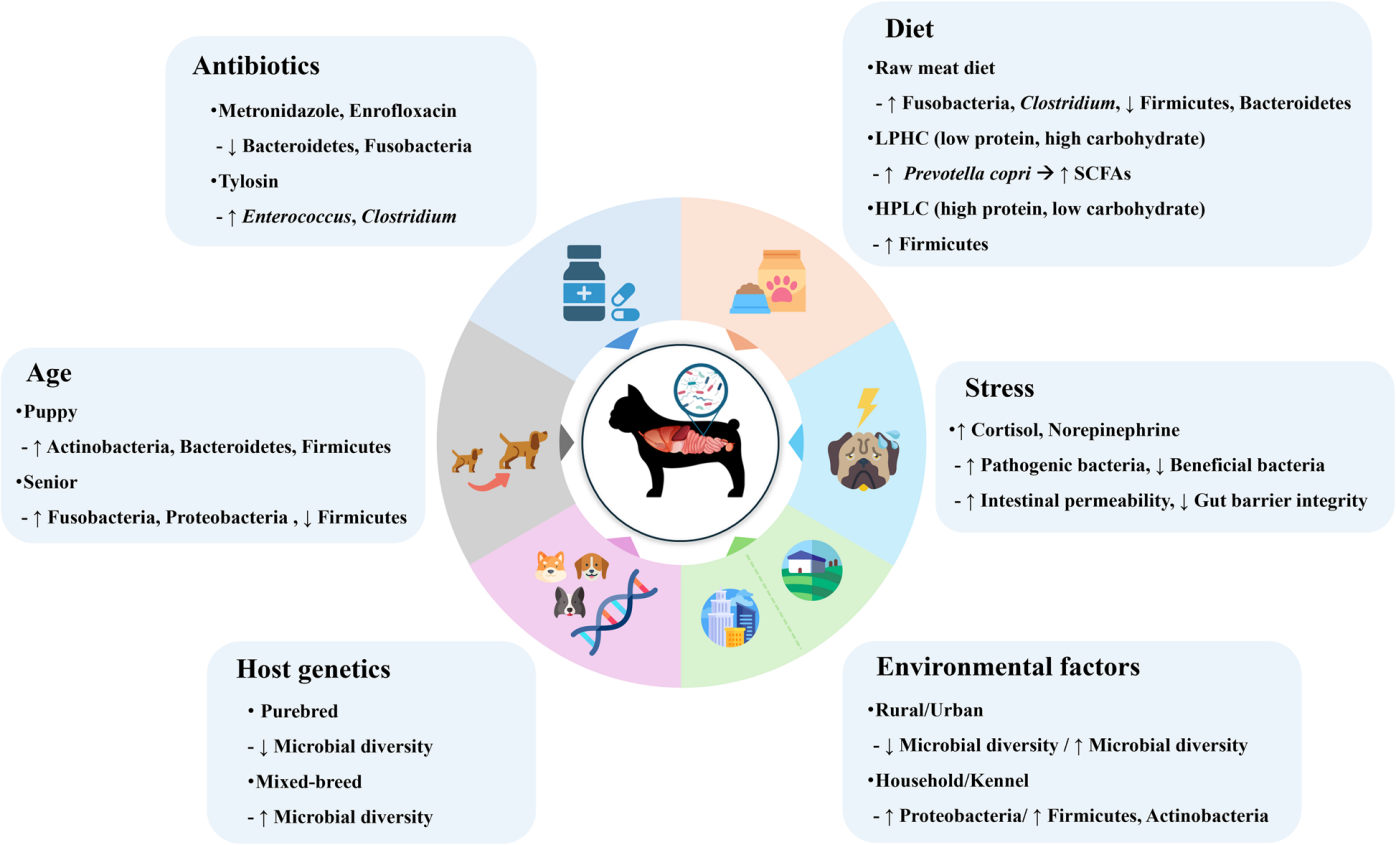
Factors influencing the gut microbiome in canines. (The figure was created using freely available images from Freepik: https://www.freepik.com/)

The balance of the gut microbiota in canines can be influenced by various factors, including diet, antibiotics, stress, and environmental conditions [15, 23, 25, 28, 29, 53, 65–71] (Fig. 2).

Physiological factors such as age and body size significantly affect the composition and diversity of the gut microbiota, playing a crucial role in canine health and metabolic functions throughout different life stages [53, 67, 68]. Diet composition is another major determinant, as variations in protein, fiber, and carbohydrate intake can substantially alter the gut microbial communities [23, 25, 65]. Additionally, antibiotics and stress can disrupt gut homeostasis by decreasing the abundance of beneficial bacteria and promoting microbial imbalances, potentially leading to digestive and immune-related disorders [15, 28–30, 69]. Environmental factors, including living conditions and hygiene levels, also influence gut microbiota diversity [70, 71]. Canines exposed to more diverse microbial environments tend to develop distinct microbial profiles [70, 71]. Understanding the correlation between these factors and the gut microbiota is essential for maintaining microbial balance. Based on this knowledge, targeted strategies, such as probiotic supplementation, dietary modifications, and stress management, can be implemented to help maintain a balanced gut microbiome and support optimal gut health in canines.

### Influence of physiological factors on the canine gut microbiome

The composition and balance of the gut microbiome in canines are significantly influenced by physiological factors such as age and body size, all of which play a crucial role in maintaining overall health and supporting metabolic functions throughout a canine’s life cycle [42, 53, 67, 68, 72, 73].

As canines age, their gut microbiome stabilizes and matures [74–77]. During the early developmental stage, bacterial diversity gradually increases, reaching an optimal balance in adulthood. However, microbial diversity tends to decline in the senior stage, potentially leading to impaired digestive function and weakened immunity [75, 76]. During the puppy stage, nutrients and lactic acid bacteria from breast milk contribute to higher proportions of Firmicutes, Bacteroidetes, and Actinobacteria, which are essential for enhancing digestive function and immune system development during this period [78]. In contrast, older canines tend to exhibit a reduction in Firmicutes and an increase in Proteobacteria [76, 79]. These microbial shifts are associated with reduced digestive efficiency and immune function, creating conditions that favor the growth of pathogenic bacteria and potentially exacerbating gut microbiome dysbiosis [76, 79, 80].

Body size affects the capacity and retention time of the digestive system, altering the gut environment, such as pH levels, which in turn impacts the composition of the intestinal microbial community [73]. Large canines have longer and more voluminous digestive tracts, allowing food to remain in the gut for a longer duration, thereby enhancing digestion and fermentation [53]. This prolonged retention increases the production of SCFAs, which lower gut pH and inhibit the growth of acid-sensitive pathogenic microbes [73, 81]. According to a study by Deschamps et al. [53], large canines have a higher proportion of Firmicutes and a lower proportion of Proteobacteria compared to small canines. This difference is attributed to the more acidic environment created by intestinal fermentation in large canines, which suppresses the growth of Proteobacteria.

In contrast, small canines have relatively shorter intestines, creating a gut environment where the intestinal pH remains closer to neutral. This condition favors the proliferation of microbiota such as Proteobacteria [53, 81]. A high proportion of Proteobacteria is closely associated with gut microbiota dysbiosis, which may negatively affect gut health [8, 42, 67].

Overall, the composition and relative abundance of the gut microbiome in canines vary significantly based on physiological factors, such as age and body size. To maintain a balanced gut microbiome, it is essential to consider these unique characteristics.

### Host genetics

The composition and balance of the canine gut microbiome are influenced by host genetic factors, with breed serving as a key indicator of genetic variation [82–85].

Reddy et al. [82] reported breed-dependent differences in gut microbial composition among small breeds such as Maltese, Poodle, and Miniature Schnauzer. Notably, Maltese dogs exhibited a significantly lower relative abundance of Firmicutes and a higher proportion of Fusobacteria compared to the other breeds. Similarly, Hu et al. [85] examined large breeds including Chinese Kunming Canine, German Shepherd, and Belgian Malinois, and observed marked differences in microbial composition at both the phylum and genus levels, despite identical environmental and dietary conditions. These findings underscore the significant role of host genetics in shaping gut microbiota across both small and large breeds. In another study by Li et al. [83], compared the gut microbiome of three working breeds including German Shepherd, Labrador Retriever, and Springer Spaniel, and found that German Shepherd exhibited significantly higher alpha diversity than the other breeds as measured by the Chao1 and Shannon indices (*P* < 0.05). These suggest that both compositional and diversity-related differences in the canine gut microbiome may be attributed to breed-specific genetic backgrounds [82, 83, 85].

Melis et al. [84] further emphasized the influence of genetic diversity on microbial balance. In the highly inbred Norwegian Lundehunds, the gut microbiome was characterized by dysbiosis with an elevated Firmicutes/Bacteroidetes ratio and overgrowth of *Streptococcus bovis*/*equinus*. In contrast, F1 and F2 crossbred generations displayed a more balanced microbiome composition, marked by a lower Firmicutes/Bacteroidetes ratio and increased abundance of health-associated genera such as *Blautia* and *Fusobacterium*. These findings suggest that increased host genetic diversity, achieved through crossbreeding, may promote a more stable and health-associated gut microbiome.

In summary, breed-specific genetic traits significantly influence the composition and diversity of the canine gut microbiome. However, comprehensive studies across a wider range of breeds are needed to fully elucidate the genetic determinants of microbiome variation in dogs.

### Diet

Among the various factors influencing the gut microbiome of canines, diet has the most consistent and significant impact throughout their lifetime. The type, form, and composition of a diet can either promote or suppress the growth of specific gut microbiota. Differences in microbiome composition associated with diet are largely attributable to variations in nutrient content, particularly the proportions of protein and carbohydrates, which play a major role in shaping the gut microbiome [23, 63].

Previous studies have demonstrated how the relative proportions of protein, carbohydrates, and fiber influence gut microbial communities [86, 87]. According to a study by Kim et al. [88], canines fed a raw meat diet exhibited increased gut microbial diversity and notable shifts in Fusobacteria abundance at the phylum level. Similarly, other studies have observed that canines fed raw meat-based diets showed increased proportions of Fusobacteria and *Clostridium*, along with decreased proportions of Firmicutes and Bacteroidetes [25, 89]. These findings align with a study by Sandri et al. [65], which found that canines consuming a high-protein diet had an increased proportion of bacteria belonging to the Fusobacteriaceae family.

In contrast, a study by Lewis et al. [23] reported that canines fed a low-protein, high-carbohydrate diet exhibited an increased proportion of bacteria associated with SCFAs production, including *Prevotella copri*, which is involved in carbohydrate fermentation. Similar changes in intestinal microflora were observed in canines that consumed prebiotics such as inulin and fructooligosaccharides. A review by Pilla and Suchodolski [24] further supported that a diet including prebiotics promotes SCFAs production and increases the proportion of beneficial bacteria such as *Bifidobacterium* and *Faecalibacterium.*

Additionally, a study by Panasevich et al. [86] found that a diet containing potato fiber may have a prebiotic effect and contribute to improving the intestinal environment of companion canines. As the concentration of potato fiber increased, the proportion of Firmicutes increased, while the proportion of Fusobacteria decreased, suggesting that the inclusion of potato fiber can influence gut microbiota composition.

Collectively, these findings emphasize the crucial role of diet in maintaining and improving gut microbiome balance in canines.

### Antibiotics

Unlike physiological factors or diet, which consistently influence the gut microbiota throughout a canine's life, antibiotics have a temporary but significant impact on the gut microbial community [15, 28, 69, 90].

While antibiotics are primarily used to suppress specific pathogenic bacteria, their excessive or prolonged use can disrupt microbial balance by indiscriminately eliminating beneficial bacterial populations, leading to dysbiosis [28, 45, 91–94]. For instance, antibiotics such as metronidazole may reduce the populations of certain microbial groups while increasing the relative abundance of *E. coli*, which can exacerbate intestinal inflammation [15].

A study by Whittemore et al. [28] demonstrated significant alterations in the canine gut microbiome following the administration of enrofloxacin and metronidazole, as assessed by 16S rRNA gene sequencing. All dogs received antibiotics for 21 d, followed by either a placebo or a synbiotic supplement. After antibiotic treatment, the abundance of *Clostridium hiranonis* (*C. hiranonis*), *Faecalibacterium*, and *Turicibacter* markedly decreased, leading to severe microbiome disruption. In the placebo group, microbial composition did not recover even after 56 days, resulting in prolonged gut dysbiosis. In contrast, partial restoration of beneficial bacteria was observed in the synbiotic group. Additionally, a decrease in alpha diversity indicated reduced microbial richness, while beta diversity analysis revealed significant differences in microbiome composition between the groups. Metabolomic analysis also showed distinct alterations in SCFAs, bile acids, and tryptophan metabolites [70]. These findings highlight the negative impact of prolonged antibiotic use on gut microbial diversity while suggesting the potential benefits of synbiotics in promoting microbiome recovery.

Similarly, research by Pilla et al. [15] demonstrated that metronidazole significantly reduced both the diversity and abundance of the canine gut microbiota. Specifically, bacteria associated with SCFAs production, anti-inflammatory activity, and immune regulation, such as Fusobacteria*, Faecalibacterium*, and *C. hiranonis*, declined substantially [15, 95].

A study by Marshell-Jones et al. [69] further investigated the impact of metronidazole on the canine gut microbiome, noting that while some beneficial bacteria increased post-treatment, overall microbial diversity decreased. This reduction in diversity was accompanied by an increase in antibiotic-resistant genera such as *Lactobacillus*, *Bifidobacterium*, and *Enterococcus*, while beneficial carbohydrate-fermenting bacteria, such as members of the Erysipelotrichaceae family, declined. Notably, gut dysbiosis persisted for 4–6 weeks after discontinuation of the antibiotic, demonstrating its prolonged effects on digestive and immune functions.

Tylosin, a macrolide antibiotic commonly used to treat chronic intermittent diarrhea and other gastrointestinal disorders in canines, has also been shown to reduce gut microbial diversity and alter the relative abundance of specific microbial taxa. Following tylosin administration, an increase in the proportion of Firmicutes and a corresponding decrease in Bacteroidetes have been observed, leading to microbiome imbalance. Additionally, tylosin has been associated with an increase in *Enterococcus* and *Clostridium* species. These alterations in the gut microbiome may also influence metabolic processes by modifying the gut metabolite profile, potentially affecting digestion and immune function [90].

In summary, antibiotic use can have profound and often adverse effects on gut microbiome balance, diversity, and associated metabolic processes. Careful antibiotic use, combined with strategies such as synbiotic supplementation, is crucial for mitigating these adverse effects and supporting the recovery of the gut ecosystem.

### Stress

Stress is a significant factor that influences the gut microbiome in companion canines. Unlike more consistent factors such as diet, which have long-term effects, stress can exert both acute and chronic impacts on the gut microbiome, depending on the nature, intensity, and duration of the stressor [29, 30, 96–98]. Acute stressors, such as environmental changes or alterations in routine, can temporarily disrupt gut microbiota composition. In contrast, chronic stress has been associated with more sustained alterations, potentially leading to dysbiosis and negatively affecting overall health [29, 97, 98].

Previous studies have shown that stress hormones like cortisol and norepinephrine can increase the virulence of certain pathogenic bacteria, such as *Salmonella enterica*, by up to tenfold [30, 96]. Excessive cortisol secretion contributes to reduced populations of beneficial bacteria, increased intestinal permeability, and overgrowth of pathogenic microbes, which can have adverse effects on canine health. Additionally, a study by Sacoor et al. [29] demonstrated that stress negatively impacts gut barrier integrity, triggering inflammatory responses and highlighting critical interactions between gut microbiota and immune responses in mental health issues such as anxiety disorders.

Recent studies have further demonstrated that stress significantly influences gut microbiota composition in canines, leading to measurable changes in microbial diversity [29, 30, 97, 98].

According to Mondo et al. [30], stressed canines exhibited a significant increase in Proteobacteria (*P* < 0.001) and a decrease in Clostridia (*P* = 0.008) compared to non-stressed controls, patterns that are often associated with dysbiosis and inflammatory responses. Furthermore, the abundance of Bacteroidaceae was significantly higher in stressed canines (*P* < 0.02) compared to the non-stressed group, suggesting a microbial imbalance caused by stress exposure.

Additionally, microbial diversity analyses revealed that α-diversity was significantly reduced in the stressed group compared to non-stressed canines (*P* < 0.05), indicating decreased microbial richness and evenness. In contrast, β-diversity analysis confirmed distinct microbial community structures between stressed and non-stressed canines (*P* < 0.01), further supporting the impact of stress on gut microbiome composition [30]. These findings suggest that stressed canines may struggle to maintain microbial diversity, fostering an environment where specific pathogenic microbes dominate [29, 30, 96–99].

Collectively, these findings emphasize the importance of stress management in maintaining the balance of the intestinal microbial community in companion canines, highlighting the need for interventions to mitigate stress-related gut dysbiosis and its associated health consequences.

### Rearing environment

The environment in which companion canines are raised significantly influences the diversity of their gut microbiome [70, 71, 100]. A study by Vilson et al. [70] compared the gut microbial diversity of puppies raised in metropolitan, small-town, and rural environments. The results revealed that puppies raised in metropolitan areas exhibited higher microbial diversity (Shannon diversity index: 3.36 ± 0.63) compared to those raised in rural (2.91 ± 0.83) or small-town environments (2.95 ± 0.81). This suggests that diverse environmental stimuli in urban settings play a crucial role in gut microbiome development. Notably, the Shannon diversity index of urban puppies was 15%–20% higher than that of puppies raised in other environments, potentially due to greater exposure to a wider range of microbial sources. These findings emphasize the need to consider environmental factors when aiming to maintain gut microbiome balance in companion canines [100, 101].

In a study by Tanprasertsuk et al. [71], significant differences in the canine gut microbiota composition were observed between groups with different housing conditions. In household environments, Proteobacteria were identified as the most abundant phylum, while Firmicutes dominated in private kennel settings, and Actinobacteria were prevalent in controlled research environments. The study also employed α-diversity and β-diversity indices to measure microbial diversity. Canines raised in private kennels exhibited significantly higher microbial diversity compared to those raised in household environments, while canines in controlled research environments showed greater α-diversity than household-raised canines. Furthermore, β-diversity analysis revealed significant structural differences in the gut microbiome among the three groups, underscoring the importance of rearing environments in maintaining gut microbiome balance [71].

However, Tanprasertsuk et al. [71] noted that dietary differences among the groups might have contributed to the observed microbiome changes. Therefore, it cannot be conclusively determined that these differences were solely due to environmental factors [24, 87, 102]. Further studies that control for diet and other confounding variables are necessary to isolate the specific impact of environmental factors on the gut microbiome [71].

In conclusion, the balance of the gut microbiome in companion canines is significantly influenced by rearing environments. A tailored approach that considers rearing environments is essential for maintaining and improving canine health. Continuous monitoring and management of environmental conditions are particularly important for sustaining gut microbiome equilibrium.

## The balance of gut microbiome and its importance in canine health and physiological functions

**Fig. 3:**
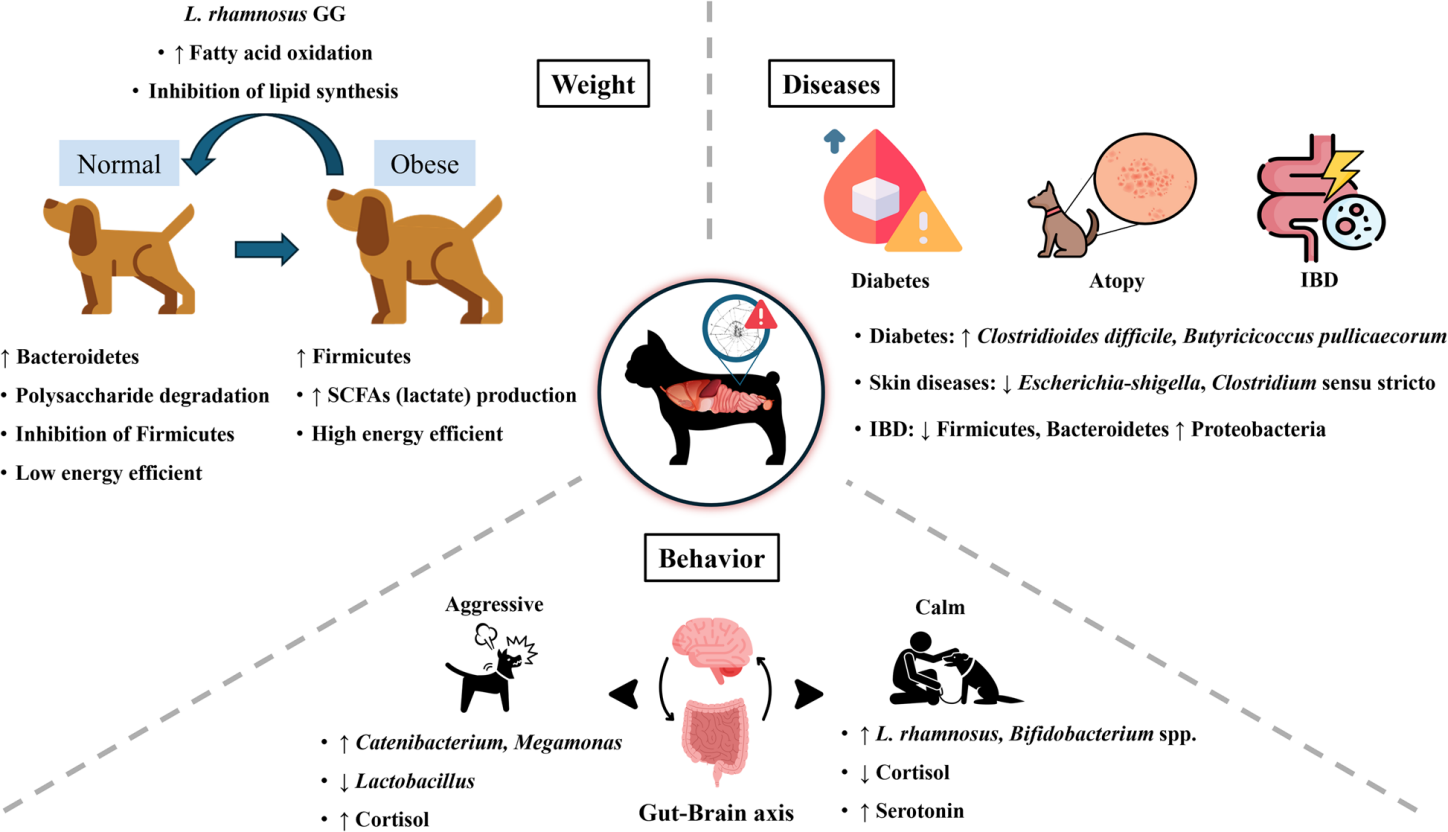
The relationship between physiological functions and the gut microbiome in canines. (The figure was created using freely available images from Freepik: https://www.freepik.com/)

The gut microbiome of canines plays a vital role in overall health, influencing key physiological functions such as digestion, immune responses, and behavioral temperament [66, 103] (Fig. 3).

Specifically, maintaining gut eubiosis is essential for inhibiting the growth of pathogenic microorganisms and regulating immune responses, thereby positively impacting overall canine health [3, 75, 86, 104]. For example, a balanced gut microbiome supports emotional stability and stress resilience by modulating the gut-brain axis, while also enhancing nutrient absorption and energy utilization [29, 96]. Therefore, understanding the gut microbiome can contribute to improving physiological functions, behavioral regulation, and the prevention and management of disease in companion canines [45]. This highlights the importance of maintaining gut eubiosis through targeted interventions such as probiotics, prebiotics, and dietary adjustments, all of which play a crucial role in enhancing the overall health and quality of life of canines.

### Weight management and gut microbiome

Maintaining a balanced gut microbiome is essential for canine weight management, as microbial composition influences metabolic processes, energy regulation, and nutrient absorption. Imbalances in the gut microbiota can lead to weight fluctuations, whereas a well-maintained microbiome supports healthier weight control [3, 9, 41, 105].

According to a previous study, obese canines showed an increased proportion of Firmicutes and a decreased proportion of Bacteroidetes compared to non-obese individuals [105]. This trend has been consistently observed in subsequent study, with a higher Firmicutes/Bacteroidetes ratio detected in obese canines, suggesting a potential link between gut microbial composition and energy extraction efficiency [106].

This association suggests that weight gain in canines may be linked to changes in microbial-mediated energy efficiency. The Firmicutes phylum is known to produce metabolites such as SCFAs during intestinal metabolism, which plays a crucial role in digestion and overall host health [3, 53, 54]. For instance, species such as *Lactobacillus acidophilus, Clostridium butyricum*, and *Oscillibacter* within Firmicutes produce lactic acid, which promotes intestinal health by lowering luminal pH, inhibiting pathogen overgrowth, and encouraging the proliferation of beneficial bacteria [104, 107–109]. Furthermore, SCFAs serve as a direct energy source for intestinal cells, enhancing nutrient utilization and overall energy extraction from the diet [107, 110].

However, excessive SCFAs production can lead to an energy surplus, contributing to increased body fat accumulation [9, 111]. This aligns with findings that a higher proportion of Firmicutes is often associated with weight gain due to enhanced energy extraction from the diet [3, 9, 41, 112–117]. In contrast, Bacteroidetes are less efficient at extracting energy from food than Firmicutes, which explains why a higher proportion of Bacteroidetes is less correlated with weight gain [9, 112]. Therefore, effective weight management in canines requires maintaining the composition and balance of the gut microbiome. The use of probiotics may aid in this process by promoting gut health and supporting a balanced microbiome [3, 15, 80].

However, the association between the Firmicutes-to-Bacteroidetes ratio and obesity is not always consistent across animal species. For example, while obese companion dogs tend to exhibit an increased Firmicutes/Bacteroidetes ratio, the opposite trend has been reported in companion cats, where obesity is associated with a significantly lower Firmicutes/Bacteroidetes ratio. These contrasting results suggest that the relationship between the Firmicutes/Bacteroidetes ratio and obesity may vary by species and does not necessarily follow a universal pattern [118].

Among probiotic strains, *Lacticaseibacillus rhamnosus* (*L*. *rhamnosus*) GG has demonstrated significant potential for weight management. According to a study by Kim et al. [119], *L. rhamnosus* GG increased adiponectin secretion, thereby activating the adenosine monophosphate-activated protein kinase (AMPK) pathway. This activation suppressed weight gain and liver fat accumulation in obese animal, suggesting its potential as a therapeutic agent for preventing metabolic syndrome [119]. Similarly, Zhang et al. [120] demonstrated that the culture supernatant of *L. rhamnosus* GG reduced hepatic fat accumulation by enhancing AMPK activation, suppressing lipid synthesis-related gene expression, and promoting fatty acid β-oxidation. These findings suggest that probiotics with similar properties to *L. rhamnosus* GG may contribute to weight management and metabolic health in canines by modulating gut microbiome composition and function. Therefore, restoring gut microbiome balance through probiotic interventions holds promises for supporting healthy weight management in canines.

### Disease and gut microbiome

#### Inflammatory bowel disease (IBD)

IBD is a chronic inflammatory condition of the GIT that is closely associated with gut microbiome imbalance [4, 11, 19, 44, 121–123].

Previous studies have reported that the small intestinal microbiome of canines with IBD differs significantly from that of healthy canines, as revealed by β-diversity analysis. Canines with IBD exhibit significantly lower species richness, indicating reduced gut microbiome diversity [5, 11]. Dysbiosis in the gut microbiome of canines with IBD can lead to a reduction in metabolic products such as SCFAs resulting in a weakened protective function of the intestinal mucosa and an increase in intestinal pH, which promotes the proliferation of pathogenic bacteria. The microbial imbalance contributes to a self-perpetuating cycle that impairs metabolic homeostasis and immune responses, promoting the chronic progression and exacerbation of IBD [123].

In particular, studies have shown that the gut microbiome of canines with IBD was characterized by a marked decrease in the phyla Firmicutes and Bacteroidetes and an increase in Proteobacteria compared to healthy canines. These shifts were often associated with the overgrowth of pathogenic bacteria such as *E. coli* [11, 121, 122]. This microbial imbalance caused by pathogenic bacteria damages intestinal epithelial cells, allowing bacterial metabolites and toxins to enter the bloodstream through a weakened intestinal barrier. This process triggers excessive immune responses, further contributing to the chronic progression and persistence of IBD [4, 19].

To address these issues, therapeutic strategies such as fecal microbiota transplantation (FMT) and probiotic administration have been explored [44, 124]. FMT involves the transplantation of gut microbiota from a healthy canine into a canine with IBD [124], and has been reported to restore gut microbiome balance, reduce gut inflammation, and improve clinical symptoms [44]. Therefore, restoring gut microbiome balance through targeted interventions could serve as a fundamental approach to improving symptoms and managing IBD in canines.

#### Diabetes

Diabetes mellitus in canines is a chronic endocrine disorder characterized by insufficient insulin production and is closely associated with gut microbiome imbalances [12, 125–128]. Previous studies have indicated that gut dysbiosis exacerbates both local and systemic inflammation, thereby exacerbating the progression of diabetes and its associated complications [12, 126–128].

For example, Jaffey et al. [125] found that diabetic canines exhibited a higher relative abundance of fungal species within the *Candida* genus compared to healthy canines. Similarly, research by Kwong et al. [126] showed an increase in *Clostridioides difficile* and *Butyricicoccus pullicaecorum* in diabetic canines, suggesting a link between metabolic dysregulation and microbial imbalance caused by the excessive proliferation of carbohydrate-dependent microbes. These findings align with previous studies investigating the gut microbiome of diabetic canines [127, 128]. On the contrary, Sharma et al. [12] reported that promoting the growth of beneficial bacteria and enhancing intestinal barrier function can help alleviate diabetes-related inflammation and improve overall health. These findings support the notion that restoring gut microbiome balance represents a promising therapeutic strategy for managing diabetes in canines and enhancing overall well-being.

#### Chronic liver disease

Chronic liver disease in canines is closely linked to disruptions in gut microbiota balance [13, 129, 130]. Habermaass et al. [13] reported that canines with chronic liver disease exhibited a marked reduction in *C. hiranonis* and *Ruminococcus faecis*, both of which play a role in bile acid metabolism and SCFAs production. Conversely, the relative abundance of potentially pathogenic genera such as *Escherichia*, *Shigella* and *Serratia* was significantly elevated in these canines. In addition, research by Cerquetella et al. [130] demonstrated that such microbial imbalances can increase intestinal permeability, allowing bacterial metabolites and endotoxins to enter the bloodstream and reach the liver, which may contribute to hepatic inflammation and liver dysfunction.

To mitigate these adverse effects, synbiotic supplementation, combining probiotics and prebiotics, has emerged as a promising intervention for canines with chronic liver disease [129, 131]. According to Habermaass et al. [131], synbiotics promoted the growth of beneficial bacteria while suppressing harmful microbial populations, thereby restoring microbial balance. This microbial modulation was associated with reduction in serum alanine aminotransferase and improvement in liver function. Additionally, Giuffrè et al. [129] suggested that synbiotics helped stabilize bile acid metabolism and enhanced the antimicrobial environment within the gut, offering further protection for hepatic function. Collectively, these findings underscore the importance of maintaining gut microbiota balance in the management of chronic liver disease and highlight the therapeutic potential of microbiome targeted interventions in improving liver health in canines [129, 131].

#### Skin diseases

Studies in humans have revealed a correlation between gut microbiota and skin health, highlighting the significance of the gut-skin axis in maintaining dermatological homeostasis [132–134]. As such, recent research in canines suggested that alterations in gut microbial composition may also influence skin health [132, 135], implying that the gut-skin axis may also play a significant role in canine skin health. Furthermore, recent study has highlighted the critical role of the gut microbiome in skin barrier function, reinforcing the strong connection between the gut and dermatologic health in companion canine [135].

For example, a study involving Finnish Lapphunds and Labrador Retrievers found that healthy canines exhibited a higher abundance of *Prevotella, Lachnospiraceae*, and *Fusobacterium*, whereas canines with atopic dermatitis showed reduced microbial diversity and richness, along with increased levels of *Escherichia* and *Shigella* [14]. Additionally, Thomsen et al. [136] demonstrated that *Fusobacterium* (20.6%) and *Megamonas* (18.4%) were abundant in healthy canines. However, these microbial groups in canines with atopic dermatitis were significantly reduced to 0.06% and 0.0003%, respectively. In contrast, *Escherichia*, *Shigella* and *Clostridium* were notably increased in canines with atopic dermatitis. These findings suggest that atopic dermatitis is associated with the overgrowth of proinflammatory gut bacteria and a significant reduction in beneficial microbial species [137].

*L. rhamnosus* has been shown to play a key role in modulating immune responses and reducing inflammation by suppressing the production of inflammatory cytokines. In chronic inflammatory diseases such as atopic dermatitis, *L. rhamnosus* activates immune cells to regulate immunoglobulin E (IgE) levels and modulates T-helper cell type 2 (Th-2) immune responses through interleukin-10 (IL-10), leading to a reduction in inflammation [135]. This process not only alleviates skin inflammation but also strengthens the skin barrier, enhancing resistance to external irritants.

These findings underscore the importance of gut microbiome balance in immune modulation and skin health. Restoring a healthy gut microbial composition may serve as an effective strategy to alleviate chronic inflammatory skin disorders such as atopic dermatitis and to support overall dermatological well-being of companion canines.

### Behavior/Temperament and gut microbiome

The gut microbiome can influence canine behavior and temperament through the gut-brain axis [14, 29, 103, 138]. According to Mondo et al. [66], aggressive canines exhibited a higher relative abundance of *Catenibacterium* and *Megamonas* compared to non-aggressive counterparts, indicating a potential gut microbiota imbalance. This dysbiosis may disrupt hormonal signaling and neural communication through the gut-brain axis, potentially exacerbating stress responses and impulsive behaviors. Similarly, Beerda et al. [139] found that gut microbiota imbalance was associated with systemic inflammation, leading to elevated cortisol levels and increased production of pro-inflammatory cytokines. These changes impair stress-regulating mechanisms and may heighten stress sensitivity and aggressive tendencies in canines. These findings suggest that gut microbiome balance plays a crucial role in behavioral regulation, stress responses, and overall temperament in canines.

Moreover, a reduction in beneficial bacteria such as *Lactobacillus* has been shown to disrupt the regulation of neurotransmitters including gamma-aminobutyric acid (GABA) and serotonin, both of which are critical for maintaining emotional stability and nervous system homeostasis [138]. The disruption in GABA expression reduces the brain's ability to mitigate stress responses, further intensifying behaviors linked to anxiety and impulsivity [66, 103].

To address these issues, the use of beneficial bacteria has been suggested as an effective intervention. For instance, *L. rhamnosus* has been reported to improve the gut microbial environment and alleviate stress responses [29, 137]. Studies have shown that *L. rhamnosus* lowered circulating cortisol levels, a key stress hormone, thereby reducing anxiety and stress responses [138, 140, 141]. These findings suggest that beneficial bacteria such as *L. rhamnosus* may contribute to neurological stability in canines.

In support of this, Hsiao et al. [103] emphasized the role of gut microbiome balance in stabilizing the nervous system, reducing anxiety, and promoting positive behavioral changes. Similarly, several studies suggested that stabilizing and maintaining gut microbiome balance can reduce stress and anxiety responses, ultimately leading to improved behavior and more stable temperament in canines [29, 137, 138]. Above all, maintaining a balanced gut microbiome is essential for regulating the gut-brain axis and enhancing nervous system function [29, 137].

## Strategies for maintaining gut microbiome balance

**Fig. 4:**
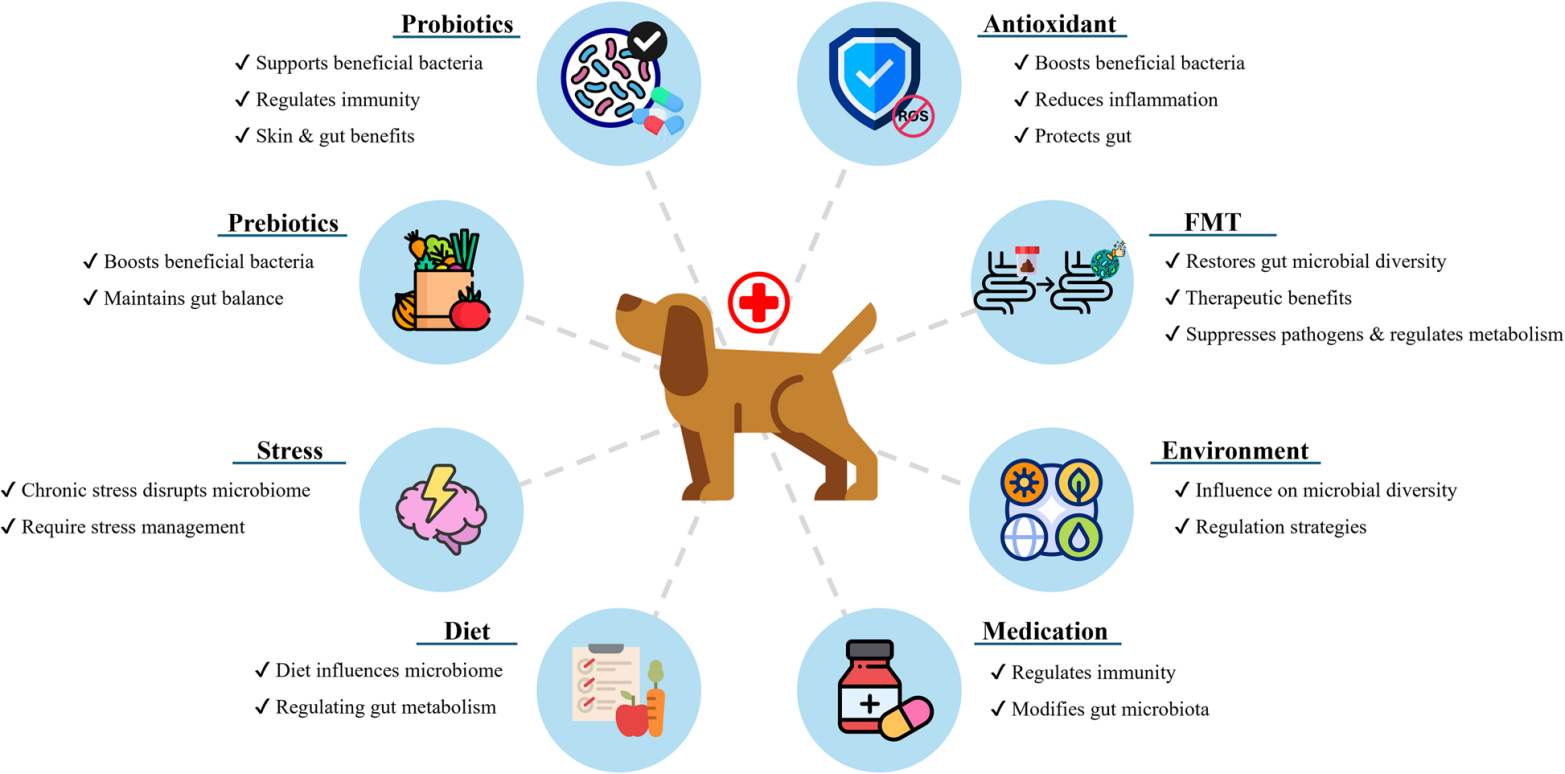
Strategies for maintaining a balanced gut microbiome in canine. (The figure was created using freely available images from Freepik: https://www.freepik.com/)

The gut microbiome of companion canines plays a crucial role in their overall health, and maintaining its balance can be achieved through various strategies (Fig. 4).

Approaches to promoting gut microbiome health include dietary adjustments, probiotic and prebiotic supplementation, appropriate medication, antioxidant use, and lifestyle modifications [15, 67, 142]. Among these strategies, probiotics are widely used to correct dysbiosis by promoting the growth of beneficial bacteria, while prebiotics serve as a nutrient source for beneficial microbes, contributing to gut microbiome homeostasis [15, 27, 143].

Probiotics are live microorganisms that, when consumed in sufficient amounts, provide health benefits to the host [144]. They enhance gut microbiota balance by promoting beneficial bacteria, inhibiting pathogenic microorganisms, and improving overall gut health. Commonly used strains such as *Lactobacillus* spp. and *Bifidobacterium* spp. have demonstrated beneficial effects on canine health [109, 142]. For example, *L. rhamnosus* has been shown to regulate immune responses and reduce inflammation by suppressing the production of proinflammatory cytokines. In chronic inflammatory diseases such as atopic dermatitis, *L. rhamnosus* activates immune cells, regulates IgE levels, and modulates Th-2 immune responses through IL-10, thereby reducing inflammatory reactions. This process alleviates skin inflammation, strengthens the skin barrier, and enhances resistance to external irritants [145].

The benefits of probiotics can be further enhanced when combined with prebiotics. Prebiotics are indigestible dietary components that promote the growth of beneficial bacteria while inhibiting harmful microbes [27, 146, 147]. For instance, chicory-derived oligosaccharides have been shown to increase the *Bifidobacterium* abundance while reducing *C. perfringens*, thereby contributing to gut microbial balance [148].

Dietary composition significantly influences gut microbiota. High-protein and high-carbohydrate diets differentially influence microbial profiles, with high-protein diets shown to increase fecal pH and alter fatty acid concentrations [27, 63, 142]. Studies also indicate that natural diets foster greater gut microbiome diversity than commercial diets [7]. Additionally, antioxidant supplementation, such as bromelain, quercetin, and lentinula edodes, has been shown to increase the proportions of *Bifidobacterium* and *Lactobacillaceae*, promoting microbial balance and reducing inflammation [7].

Certain medications can positively influence the gut microbiome [136, 149]. For example, oclacitinib, a Janus kinase inhibitor used in canine atopic dermatitis reduces inflammation by inhibiting cytokine signaling. It has also been shown to increase the abundance of *Fusobacterium* while decreasing the prevalence of inflammatory microbes such as *Escherichia*, *Shigella* and *Clostridium* in canines with atopic dermatitis [136]. However, while antibiotics are effective in eliminating pathogenic microorganisms, they can also disrupt the gut microbiome by depleting beneficial bacteria. Therefore, supplementing with probiotics following antibiotic treatment is crucial to restoring microbial balance and preventing dysbiosis [148].

FMT has emerged as a promising intervention for restoring gut microbiome diversity and suppressing the overgrowth of harmful microorganisms [44, 150]. This technique involves transplanting fecal microbiota from a healthy donor into a canine with gut dysbiosis. Research suggests that FMT is particularly effective for conditions such as IBD, metabolic disorders, and chronic diarrhea, which are difficult to manage with conventional treatments [150]. By reintroducing a diverse microbial community, FMT helps regulate host metabolism and immune functions, restoring gut microbiome balance.

In summary, maintaining gut microbiome balance in companion canines involves a multifaceted approach, including probiotic and prebiotic supplementation to promote the growth of beneficial bacteria, antioxidant supplementation to support microbial stability, dietary adjustments tailored to the canine’s health needs, and stress reduction to support gut health. Implementing these strategies helps preserve microbial homeostasis, thereby improving overall health, immune regulation, and well-being in companion canines.

## Conclusion

This review highlights the connection between the canine gut microbiome and various aspects of overall health, including digestion, metabolism, immune regulation, behavior, temperament, and skin health. The canine gut microbiome consists of a diverse array of bacterial strains that form a complex ecosystem interacting closely with the host to maintain health and physiological homeostasis. However, the canine gut microbiome can be influenced by multiple factors, such as age, physiology, living environment, diet, antibiotic use, stress, and host genetics. If these factors are not properly managed, microbial imbalance (dysbiosis) may occur, potentially exacerbating health issues such as IBD, diabetes, dermatological disorders, and behavioral changes. Therefore, effective gut microbiome management is essential for optimizing physiological functions, ultimately enhancing the health and quality of life of companion canines. Strategies to promote gut microbiome health, such as dietary adjustments, administration of probiotics and prebiotics, appropriate medication, and lifestyle modifications are increasingly being implemented.

Overall, this review provides insights into the structure and function of the canine gut microbiome, emphasizing its role in health and the factors that influence its balance and maintenance.

## Data Availability

Not applicable.

## Abbreviations

AMPK: Adenosine monophosphate-activated protein kinase
*C. **hiranonis*: *Clostridium hiranonis*
CMDI: Canine microbiota dysbiosis index
*C. ** perfringens*: *Clostridium perfringens*
*E. coli*: *Escherichia coli*
FMT: Fecal microbiota transplantation
GABA: Gamma-aminobutyric acid
GIT: Gastrointestinal tract
IBD: Inflammatory bowel disease
IgE: Immunoglobulin E
IL-10: Interleukin-10
*L*. * rhamnosus*: *Lacticaseibacillus rhamnosus*
SCFAs: Short-chain fatty acids
Th-2: T-helper cell type 2

## References

[1] Cohen SP. Can pets function as family members? West J Nurs Res. 2002;24(6):621–38. 10.1177/019394502320555386. 12365764 10.1177/019394502320555386

[2] Grzeskowiak L, Endo A, Beasley S, Salminen S. Microbiota and probiotics in canine and feline welfare. Anaerobe. 2015;34:14–23. 10.1016/j.anaerobe.2015.04.002. 25863311 10.1016/j.anaerobe.2015.04.002 PMC7111060

[3] Hooda S, Minamoto Y, Suchodolski JS, Swanson KS. Current state of knowledge: the canine gastrointestinal microbiome. Anim Health Res Rev. 2012;13(1):78–88. 10.1017/s1466252312000059. 22647637 10.1017/S1466252312000059

[4] Suchodolski JS, Markel ME, Garcia-Mazcorro JF, Unterer S, Heilmann RM, Dowd SE, et al. The fecal microbiome in dogs with acute diarrhea and idiopathic inflammatory bowel disease. PLoS ONE. 2012;7(12):e51907. 10.1371/journal.pone.0051907. 23300577 10.1371/journal.pone.0051907 PMC3530590

[5] Xenoulis PG, Palculict B, Allenspach K, Steiner JM, Van House AM, Suchodolski JS. Molecular-phylogenetic characterization of microbial communities imbalances in the small intestine of dogs with inflammatory bowel disease. Fems Microbiol Ecol. 2008;66(3):579–89. 10.1111/j.1574-6941.2008.00556.x. 18647355 10.1111/j.1574-6941.2008.00556.x

[6] Pereira AM, Clemente A. Dogs’ microbiome from tip to toe. Top Companion Anim Med. 2021;45:100584. 10.1016/j.tcam.2021.100584. 34509665 10.1016/j.tcam.2021.100584

[7] Marelli SP, Fusi E, Giardini A, Martino PA, Polli M, Bruni N, et al. Effects of probiotic lactobacillus acidophilus D2/CSL (CECT 4529) on the nutritional and health status of boxer dogs. Vet Rec. 2020;187(4):e28-e. 10.1136/vr.105434. 32179577 10.1136/vr.105434 PMC7456698

[8] Garcia-Mazcorro JF, Dowd SE, Poulsen J, Steiner JM, Suchodolski JS. Abundance and short-term temporal variability of fecal microbiota in healthy dogs. Microbiologyopen. 2012;1(3):340–7. 10.1002/mbo3.36. 23170232 10.1002/mbo3.36 PMC3496977

[9] Turnbaugh PJ, Ley RE, Mahowald MA, Magrini V, Mardis ER, Gordon JI. An obesity-associated gut microbiome with increased capacity for energy harvest. Nature. 2006;444(7122):1027–31. 10.1038/nature05414. 17183312 10.1038/nature05414

[10] Ley RE, Bäckhed F, Turnbaugh P, Lozupone CA, Knight RD, Gordon JI. Obesity alters gut microbial ecology. Proc Natl Acad Sci U S A. 2005;102(31):11070–5. 10.1073/pnas.0504978102. 16033867 10.1073/pnas.0504978102 PMC1176910

[11] Vázquez-Baeza Y, Hyde ER, Suchodolski JS, Knight R. Dog and human inflammatory bowel disease rely on overlapping yet distinct dysbiosis networks. Nat Microbiol. 2016;1:16177. 10.1038/nmicrobiol.2016.177. 27694806 10.1038/nmicrobiol.2016.177

[12] Sharma S, Tripathi P. Gut microbiome and type 2 diabetes: where we are and where to go? J Nutr Biochem. 2019;63:101–8. 10.1016/j.jnutbio.2018.10.003. 30366260 10.1016/j.jnutbio.2018.10.003

[13] Habermaass V, Olivero D, Gori E, Mariti C, Longhi E, Marchetti V. Intestinal microbiome in dogs with chronic hepatobiliary disease: can we talk about the gut–liver axis? Animals. 2023;13(20):3174. 10.3390/ani13203174. 37893898 10.3390/ani13203174 PMC10603696

[14] Kirchoff NS, Udell MA, Sharpton TJ. The gut microbiome correlates with conspecific aggression in a small population of rescued dogs (*Canis familiaris*). PeerJ. 2019;7:e6103. 10.7287/peerj.preprints.26990v1. 30643689 10.7717/peerj.6103 PMC6330041

[15] Pilla R, Gaschen FP, Barr JW, Olson E, Honneffer J, Guard BC, et al. Effects of metronidazole on the fecal microbiome and metabolome in healthy dogs. J Vet Intern Med. 2020;34(5):1853–66. 10.1111/jvim.15871. 32856349 10.1111/jvim.15871 PMC7517498

[16] Keum GB, Pandey S, Kim ES, Doo H, Kwak J, Ryu S, et al. Understanding the diversity and roles of the ruminal microbiome. J Microbiol. 2024;63:217–30. 10.1007/s12275-024-00121-4. 10.1007/s12275-024-00121-4 38662310

[17] Robinson CJ, Bohannan BJM, Young VB. From structure to function: the ecology of host-associated microbial communities. Microbiol Mol Biol Rev. 2010;74(3):453–76. 10.1128/MMBR.00014-10. 20805407 10.1128/MMBR.00014-10 PMC2937523

[18] Blake AB, Suchodolski JS. Importance of gut microbiota for the health and disease of dogs and cats. Anim Front. 2016;6(3):37–42. 10.2527/af.2016-0032.

[19] AlShawaqfeh MK, Wajid B, Minamoto Y, Markel M, Lidbury JA, Steiner JM, et al. A dysbiosis index to assess microbial changes in fecal samples of dogs with chronic inflammatory enteropathy. Fems Microbiol Ecol. 2017;93(11):fix136. 10.1093/femsec/fix136. 10.1093/femsec/fix136 29040443

[20] Suchodolski JS. Analysis of the gut microbiome in dogs and cats. Vet Clin Pathol. 2022;50(Suppl 1):6–17. 10.1111/vcp.13031. 34514619 10.1111/vcp.13031 PMC9292158

[21] Sung C-H, Marsilio S, Chow B, Zornow KA, Slovak JE, Pilla R, et al. Dysbiosis index to evaluate the fecal microbiota in healthy cats and cats with chronic enteropathies. J Feline Med Surg. 2022;24(6):e1–12. 10.1177/1098612x221077876. 35266809 10.1177/1098612X221077876 PMC9160961

[22] Laboratory TAMUG. Canine microbiota dysbiosis index 2025. Available from: https://vetmed.tamu.edu/gilab/service/assays/canine-microbiota-dysbiosis-index/.

[23] Lewis S, Nash A, Li Q, Ahn T-H. Comparison of 16S and whole genome dog microbiomes using machine learning. BioData Min. 2021;14(1):41. 10.1186/s13040-021-00270-x. 34419136 10.1186/s13040-021-00270-x PMC8379800

[24] Pilla R, Suchodolski JS. The gut microbiome of dogs and cats, and the influence of diet. Vet Clin Small Anim Pract. 2021;51(3):605–21. 10.1016/j.cvsm.2021.01.002. 10.1016/j.cvsm.2021.01.002 33653538

[25] Schmidt M, Unterer S, Suchodolski JS, Honneffer JB, Guard BC, Lidbury JA, et al. The fecal microbiome and metabolome differs between dogs fed bones and raw food (BARF) diets and dogs fed commercial diets. PLoS ONE. 2018;13(8):e0201279. 10.1371/journal.pone.0201279. 30110340 10.1371/journal.pone.0201279 PMC6093636

[26] Grześkowiak Ł, Endo A, Beasley S, Salminen S. Microbiota and probiotics in canine and feline welfare. Anaerobe. 2015;34:14–23. 10.1016/j.anaerobe.2015.04.002. 25863311 10.1016/j.anaerobe.2015.04.002 PMC7111060

[27] Ciaravolo S, Martínez-López LM, Allcock RJ, Woodward AP, Mansfield C. Longitudinal survey of fecal microbiota in healthy dogs administered a commercial probiotic. Front Vet Sci. 2021;8:664318. 10.3389/fvets.2021.664318. 34235200 10.3389/fvets.2021.664318 PMC8255976

[28] Whittemore JC, Price JM, Moyers T, Suchodolski JS. Effects of synbiotics on the fecal microbiome and metabolomic profiles of healthy research dogs administered antibiotics: a randomized, controlled trial. Front Vet Sci. 2021;8:665713. 10.3389/fvets.2021.665713. 34124225 10.3389/fvets.2021.665713 PMC8187564

[29] Sacoor C, Marugg JD, Lima NR, Empadinhas N, Montezinho L. Gut-brain axis impact on canine anxiety disorders: new challenges for behavioral veterinary medicine. Vet Med Int. 2024;2024:2856759. 10.1155/2024/2856759. 38292207 10.1155/2024/2856759 PMC10827376

[30] Mondo E, De Cesare A, Manfreda G, Sala C, Cascio G, Accorsi PA, et al. Depression and microbiome—study on the relation and contiguity between dogs and humans. Appl Sci. 2020;10(2):573. 10.3390/app10020573.

[31] Tannock GW. Microecology of the gastrointestinal tract in relation to lactic acid bacteria. Int Dairy J. 1995;5(8):1059–70. 10.1016/0958-6946(95)00043-7.

[32] Walter J. Ecological role of lactobacilli in the gastrointestinal tract: implications for fundamental and biomedical research. Appl Environ Microbiol. 2008;74(16):4985–96. 10.1128/AEM.00753-08. 18539818 10.1128/AEM.00753-08 PMC2519286

[33] Lin CY, Cross TL, Swanson KS. Comparison of mucosal microbiota populations across the gastrointestinal tract of healthy dogs. Anim Microbiome. 2025;7:2. 10.1186/s42523-024-00368-7. 39762940 10.1186/s42523-024-00368-7 PMC11702091

[34] Balakrishnan B, Luckey D, Bodhke R, Chen J, Marietta E, Jeraldo P, et al. Prevotella histicola protects from arthritis by expansion of Allobaculum and augmenting butyrate production in humanized mice. Front Immunol. 2021;12:609644. 10.3389/fimmu.2021.609644. 34017324 10.3389/fimmu.2021.609644 PMC8130672

[35] Oba PM, Kelly J, Kostiuk D, Swanson KS. Effects of weight loss and feeding specially formulated diets on the body composition, blood metabolite profiles, voluntary physical activity, and fecal metabolites and microbiota of obese dogs. J Anim Sci. 2023;101:skad073. 10.1093/jas/skad073. 36879442 10.1093/jas/skad073 PMC10083730

[36] Moon CD, Young W, Maclean PH, Cookson AL, Bermingham EN. Metagenomic insights into the roles of Proteobacteria in the gastrointestinal microbiomes of healthy dogs and cats. Microbiologyopen. 2018;7(5):e00677. 10.1002/mbo3.677. 29911322 10.1002/mbo3.677 PMC6182564

[37] Suchodolski JS, Dowd SE, Westermarck E, Steiner JM, Wolcott RD, Spillmann T, et al. The effect of the macrolide antibiotic tylosin on microbial diversity in the canine small intestine as demonstrated by massive parallel 16S rRNA gene sequencing. BMC Microbiol. 2009;9:210. 10.1186/1471-2180-9-210. 19799792 10.1186/1471-2180-9-210 PMC2759960

[38] Hand D, Wallis C, Colyer A, Penn CW. Pyrosequencing the canine faecal microbiota: breadth and depth of biodiversity. PLoS ONE. 2013;8(1):e53115. 10.1371/journal.pone.0053115. 23382835 10.1371/journal.pone.0053115 PMC3561364

[39] Middelbos IS, Vester Boler BM, Qu A, White BA, Swanson KS, Fahey GC Jr. Phylogenetic characterization of fecal microbial communities of dogs fed diets with or without supplemental dietary fiber using 454 pyrosequencing. PLoS ONE. 2010;5(3):e9768. 10.1371/journal.pone.0009768. 20339542 10.1371/journal.pone.0009768 PMC2842427

[40] You I, Kim MJ. Comparison of gut microbiota of 96 healthy dogs by individual traits: breed, age, and body condition score. Animals. 2021;11(8):2432. 10.3390/ani11082432. 34438891 10.3390/ani11082432 PMC8388711

[41] Schmitz S, Suchodolski J. Understanding the canine intestinal microbiota and its modification by pro-, pre- and synbiotics–what is the evidence? Vet Med Sci. 2016;2(2):71–94. 10.1002/vms3.17. 29067182 10.1002/vms3.17 PMC5645859

[42] Suchodolski JS. Companion animals symposium: microbes and gastrointestinal health of dogs and cats. J Anim Sci. 2011;89(5):1520–30. 10.2527/jas.2010-3377. 21075970 10.2527/jas.2010-3377 PMC7199667

[43] Alon-Maimon T, Mandelboim O, Bachrach G. Fusobacterium nucleatum and cancer. Periodontol 2000. 2022;89(1):166–80. 10.1111/prd.12426. 35244982 10.1111/prd.12426 PMC9315032

[44] Niina A, Kibe R, Suzuki R, Yuchi Y, Teshima T, Matsumoto H, et al. Fecal microbiota transplantation as a new treatment for canine inflammatory bowel disease. Biosci Microbiota Food Health. 2021;40(2):98–104. 10.12938/bmfh.2020-049. 33996366 10.12938/bmfh.2020-049 PMC8099633

[45] Pilla R, Suchodolski JS. The role of the canine gut microbiome and metabolome in health and gastrointestinal disease. Front Vet Sci. 2020;6:498. 10.3389/fvets.2019.00498. 31993446 10.3389/fvets.2019.00498 PMC6971114

[46] Sacchettino L, Costanzo M, Veneruso I, D’Argenio V, Mayer M, Napolitano F, et al. Altered microbiome and metabolome profiling in fearful companion dogs: an exploratory study. PLoS ONE. 2025;20(1):e0315374. 10.1371/journal.pone.0315374. 39813205 10.1371/journal.pone.0315374 PMC11734960

[47] Bamberger T, Muller E, Algavi YM, Greenier A, Adjangba C, Slikas E, et al. Mapping the canine microbiome: insights from the dog aging project. bioRxiv. 2024:2024.12.02.625632. 10.1101/2024.12.02.625632.

[48] Reddy KE, Kim H-R, Jeong JY, So K-M, Lee S, Ji SY, et al. Impact of breed on the fecal microbiome of dogs under the same dietary condition. J Microbiol Biotechnol. 2019. 10.4014/jmb.1906.06048. 31601060 10.4014/jmb.1906.06048

[49] Salas-Mani A, Jeusette I, Castillo I, Manuelian CL, Lionnet C, Iraculis N, et al. Fecal microbiota composition changes after a BW loss diet in Beagle dogs. J Anim Sci. 2018;96(8):3102–11. 10.1093/jas/sky193. 29790949 10.1093/jas/sky193 PMC6095273

[50] Herstad KMV, Moen AEF, Gaby JC, Moe L, Skancke E. Characterization of the fecal and mucosa-associated microbiota in dogs with colorectal epithelial tumors. PLoS One. 2018;13(5):e0198342. 10.1371/journal.pone.0198342. 29852000 10.1371/journal.pone.0198342 PMC5979030

[51] Minamoto Y, Dhanani N, Markel ME, Steiner JM, Suchodolski JS. Prevalence of *Clostridium perfringens, Clostridium perfringens* enterotoxin and dysbiosis in fecal samples of dogs with diarrhea. Vet Microbiol. 2014;174(3–4):463–73. 10.1016/j.vetmic.2014.10.005. 25458422 10.1016/j.vetmic.2014.10.005

[52] Beloshapka AN, Dowd SE, Suchodolski JS, Steiner JM, Duclos L, Swanson KS. Fecal microbial communities of healthy adult dogs fed raw meat-based diets with or without inulin or yeast cell wall extracts as assessed by 454 pyrosequencing. Fems Microbiol Ecol. 2013;84(3):532–41. 10.1111/1574-6941.12081. 23360519 10.1111/1574-6941.12081

[53] Deschamps C, Humbert D, Zentek J, Denis S, Priymenko N, Apper E, et al. From Chihuahua to Saint-Bernard: how did digestion and microbiota evolve with dog sizes. Int J Biol Sci. 2022;18(13):5086. 10.7150/ijbs.72770. 35982892 10.7150/ijbs.72770 PMC9379419

[54] Garrigues Q, Apper E, Chastant S, Mila H. Gut microbiota development in the growing dog: a dynamic process influenced by maternal, environmental and host factors. Front Vet Sci. 2022;9:964649. 10.3389/fvets.2022.964649. 36118341 10.3389/fvets.2022.964649 PMC9478664

[55] Liu X, Mao B, Gu J, Wu J, Cui S, Wang G, et al. Blautia—a new functional genus with potential probiotic properties? Gut microbes. 2021;13(1):1875796. 10.1080/19490976.2021.1875796. 33525961 10.1080/19490976.2021.1875796 PMC7872077

[56] Guo P, Zhang K, Ma X, He P. Clostridium species as probiotics: potentials and challenges. J Anim Sci Biotechnol. 2020;11:24. 10.1186/s40104-019-0402-1. 32099648 10.1186/s40104-019-0402-1 PMC7031906

[57] Betancur-Murillo CL, Aguilar-Marín SB, Jovel J. *Prevotella*: a key player in ruminal metabolism. Microorganisms. 2022;11(1):1. 10.3390/microorganisms11010001. 36677293 10.3390/microorganisms11010001 PMC9866204

[58] Singh RP. Glycan utilisation system in *Bacteroides* and *Bifidobacteria* and their roles in gut stability and health. Appl Microbiol Biotechnol. 2019;103(18):7287–315. 10.1007/s00253-019-10012-z. 31332487 10.1007/s00253-019-10012-z

[59] Ma X, Lazarowski L, Zhang Y, Krichbaum S, Smith JG, Zheng J, et al. Associations between memory performance and *Bifidobacterium pseudolongum* abundance in the canine gut microbiome. Iscience. 2024;27(5):109611. 10.1016/j.isci.2024.109611. 38638561 10.1016/j.isci.2024.109611 PMC11024906

[60] Diether NE, Willing BP. Microbial fermentation of dietary protein: an important factor in diet-microbe-host interaction. Microorganisms. 2019;7(1):19. 10.3390/microorganisms7010019. 30642098 10.3390/microorganisms7010019 PMC6352118

[61] Betancur-Murillo C, Aguilar-Marin S, Jovel J. Prevotella: a key player in ruminal metabolism. Microorganisms. 2022;11:1. 10.3390/microorganisms11010001. 36677293 10.3390/microorganisms11010001 PMC9866204

[62] Palmqvist H. Whole-grain cereals in dog food: effects on metabolism and gut microbiota. 2023. 10.54612/a.1cn95jv40c.

[63] Li Q, Lauber CL, Czarnecki-Maulden G, Pan Y, Hannah SS. Effects of the dietary protein and carbohydrate ratio on gut microbiomes in dogs of different body conditions. mBio. 2017;8(1):e01703-16. 10.1128/mBio.01703-16. 28119466 10.1128/mBio.01703-16 PMC5263242

[64] Ritchie LE, Burke KF, Garcia-Mazcorro JF, Steiner JM, Suchodolski JS. Characterization of fecal microbiota in cats using universal 16S rRNA gene and group-specific primers for *Lactobacillus* and *Bifidobacterium* spp. Vet Microbiol. 2010;144(1–2):140–6. 10.1016/j.vetmic.2009.12.045. 20092970 10.1016/j.vetmic.2009.12.045

[65] Sandri M, Dal Monego S, Conte G, Sgorlon S, Stefanon B. Raw meat based diet influences faecal microbiome and end products of fermentation in healthy dogs. BMC Vet Res. 2016;13:65. 10.1186/s12917-017-0981-z. 10.1186/s12917-017-0981-z PMC5331737 28245817

[66] Mondo E, Barone M, Soverini M, D’amico F, Cocchi M, Petrulli C, et al. Gut microbiome structure and adrenocortical activity in dogs with aggressive and phobic behavioral disorders. Heliyon. 2020;6(1):e03311. 10.1016/j.heliyon.2020.e03311. 32021942 10.1016/j.heliyon.2020.e03311 PMC6994854

[67] Suchodolski JS. The intestinal microbiome in canine chronic enteropathy and implications for extraintestinal disorders. Adv Small Anim Care. 2020;1:101–10. 10.1016/j.yasa.2020.07.008.

[68] Hu Q, Cheng L, Cao X, Shi F, Ma Y, Mo L, et al. Comparative analysis of gut microbiota of Chinese Kunming dog, German Shepherd dog, and Belgian Malinois dog. J Vet Sci. 2024;25(6):e85. 10.4142/jvs.24181. 39608779 10.4142/jvs.24181 PMC11611487

[69] Marshall-Jones ZV, Patel KV, Castillo-Fernandez J, Lonsdale ZN, Haydock R, Staunton R, et al. Conserved signatures of the canine faecal microbiome are associated with metronidazole treatment and recovery. Sci Rep. 2024;14:5277. 10.1038/s41598-024-51338-7. 38438389 10.1038/s41598-024-51338-7 PMC10912219

[70] Vilson Å, Ramadan Z, Li Q, Hedhammar Å, Reynolds A, Spears J, et al. Disentangling factors that shape the gut microbiota in German Shepherd dogs. PLoS One. 2018;13(3):e0193507. 10.1371/journal.pone.0193507. 29570709 10.1371/journal.pone.0193507 PMC5865712

[71] Tanprasertsuk J, Jones R, Tate D, Oba P, Belchik S, Swanson K, et al. Beagle dogs living in household or controlled environments show different gut microbiome profiles when fed an identical diet or diet type. Research Square. 2023. 10.21203/rs.3.rs-3167499/v1.

[72] Middleton RP, Lacroix S, Scott-Boyer MP, Dordevic N, Kennedy AD, Slusky AR, et al. Metabolic differences between dogs of different body sizes. J Nutr Metab. 2017;2017:4535710. 10.1155/2017/4535710. 29225968 10.1155/2017/4535710 PMC5684564

[73] Deschamps C, Humbert D, Chalancon S, Achard C, Apper E, Denis S, et al. Large intestinal nutritional and physicochemical parameters from different dog sizes reshape canine microbiota structure and functions in vitro. Bioengineered. 2024;15(1):2325713. 10.1080/21655979.2024.2325713. 38471972 10.1080/21655979.2024.2325713 PMC10936688

[74] Guard BC, Mila H, Steiner JM, Mariani C, Suchodolski JS, Chastant-Maillard S. Characterization of the fecal microbiome during neonatal and early pediatric development in puppies. PLoS One. 2017;12(4):e0175718. 10.1371/journal.pone.0175718. 28448583 10.1371/journal.pone.0175718 PMC5407640

[75] Hilmersson A, Sigmundsdóttir G, Vilde BS. The juvenile canine intestinal microbiota: development from birth to 12 weeks of age. Norwegian University of Life Sciences; 2020. https://hdl.handle.net/11250/2719243.

[76] Kubinyi E, Bel Rhali S, Sándor S, Szabó A, Felföldi T. Gut microbiome composition is associated with age and memory performance in pet dogs. Animals. 2020;10(9):1488. 10.3390/ani10091488. 32846928 10.3390/ani10091488 PMC7552338

[77] Bertero A, Banchi P, Del Carro A, Corrò M, Colitti B, Van Soom A, et al. Meconium microbiota in naturally delivered canine puppies. BMC Vet Res. 2024;20:363. 10.1186/s12917-024-04225-2. 39135043 10.1186/s12917-024-04225-2 PMC11318152

[78] Boucard AS, Thomas M, Lebon W, Polack B, Florent I, Langella P, et al. Age and *Giardia intestinalis* infection impact canine gut microbiota. Microorganisms. 2021;9(9):1862. 10.3390/microorganisms9091862. 34576757 10.3390/microorganisms9091862 PMC8469385

[79] Mizukami K, Uchiyama J, Igarashi H, Murakami H, Osumi T, Shima A, et al. Age-related analysis of the gut microbiome in a purebred dog colony. FEMS Microbiol Lett. 2019;366(8):fnz095. 10.1093/femsle/fnz095. 31049554 10.1093/femsle/fnz095

[80] Gebreselassie EE, Jackson MI, Yerramilli M, Jewell DE. Anti-aging food that improves markers of health in senior dogs by modulating gut microbiota and metabolite profiles. BioRxiv. 2018:324327. 10.1101/324327.

[81] Saarela M, Mogensen G, Fonden R, Mättö J, Mattila-Sandholm T. Probiotic bacteria: safety, functional and technological properties. J Biotechnol. 2000;84(3):197–215. 10.1016/S0168-1656(00)00375-8. 11164262 10.1016/s0168-1656(00)00375-8

[82] Reddy KE, Kim HR, Jeong JY, So KM, Lee S, Ji SY, et al. Impact of breed on the fecal microbiome of dogs under the same dietary condition. J Microbiol Biotechnol. 2019;29(12):1947–56. 10.4014/jmb.1906.06048. 31601060 10.4014/jmb.1906.06048

[83] Li Z, Sun Q, Li Y, Guan Z, Wei J, Li B, et al. Analysis and comparison of gut microbiome in young detection dogs. Front Microbiol. 2022;13:872230. 10.3389/fmicb.2022.872230. 35516435 10.3389/fmicb.2022.872230 PMC9063727

[84] Melis C, Billing AM, Wold PA, Ludington WB. Gut microbiome dysbiosis is associated with host genetics in the Norwegian Lundehund. Front Microbiol. 2023;14:1209158. 10.3389/fmicb.2023.1209158. 37405168 10.3389/fmicb.2023.1209158 PMC10315540

[85] Hu Q, Cheng L, Cao X, Shi F, Ma Y, Mo L, et al. Comparative analysis of the gut microbiota of the Chinese Kunming dog, German Shepherd, and Belgian Malinois. 2024. 10.21203/rs.3.rs-4288714/v1. 10.4142/jvs.24181 PMC11611487 39608779

[86] Panasevich MR, Kerr KR, Dilger RN, Fahey GC Jr, Guérin-Deremaux L, Lynch GL, et al. Modulation of the faecal microbiome of healthy adult dogs by inclusion of potato fibre in the diet. Br J Nutr. 2015;113(1):125–33. 10.1017/s0007114514003274. 25418803 10.1017/S0007114514003274

[87] Martínez-López LM, Pepper A, Pilla R, Woodward AP, Suchodolski JS, Mansfield C. Effect of sequentially fed high protein, hydrolyzed protein, and high fiber diets on the fecal microbiota of healthy dogs: a cross-over study. Anim Microbiome. 2021;3:42. 10.1186/s42523-021-00101-8. 34116725 10.1186/s42523-021-00101-8 PMC8194187

[88] Kim J, An J-U, Kim W, Lee S, Cho S. Differences in the gut microbiota of dogs (*Canis lupus familiaris*) fed a natural diet or a commercial feed revealed by the Illumina MiSeq platform. Gut Pathog. 2017;9:68. 10.1186/s13099-017-0218-5. 29201150 10.1186/s13099-017-0218-5 PMC5697093

[89] Bermingham EN, Maclean P, Thomas DG, Cave NJ, Young W. Key bacterial families (Clostridiaceae, Erysipelotrichaceae and Bacteroidaceae) are related to the digestion of protein and energy in dogs. PeerJ. 2017;5:e3019. 10.7717/peerj.3019. 28265505 10.7717/peerj.3019 PMC5337088

[90] Deschamps C, Apper E, Brun M, Durif C, Denis S, Humbert D, et al. Development of a new antibiotic-induced dysbiosis model of the canine colonic microbiota. Int J Antimicrob Agents. 2024;63(4):107102. 10.1016/j.ijantimicag.2024.107102. 38325721 10.1016/j.ijantimicag.2024.107102

[91] Grønvold AM, L’Abée-Lund TM, Sørum H, Skancke E, Yannarell AC, Mackie RI. Changes in fecal microbiota of healthy dogs administered amoxicillin. FEMS Microbiol Ecol. 2010;71(2):313–26. 10.1111/j.1574-6941.2009.00808.x. 20002181 10.1111/j.1574-6941.2009.00808.x

[92] Manchester AC, Webb CB, Blake AB, Sarwar F, Lidbury JA, Steiner JM, et al. Long-term impact of tylosin on fecal microbiota and fecal bile acids of healthy dogs. J Vet Intern Med. 2019;33(6):2605–17. 10.1111/jvim.15635. 31674054 10.1111/jvim.15635 PMC6872867

[93] Lucchetti B, Lane SL, Koenig A, Good J, Suchodolski JS, Brainard BM. Effects of a perioperative antibiotic and veterinary probiotic on fecal dysbiosis index in dogs. Can Vet J. 2021;62(3):240–6. 33692578 PMC7877677

[94] Pandey S, Doo H, Keum GB, Kim ES, Kwak J, Ryu S, et al. Antibiotic resistance in livestock, environment and humans: one health perspective. J Anim Sci Technol. 2024;66(2):266. 10.5187/jast.2023.e129. 38628683 10.5187/jast.2023.e129 PMC11016740

[95] Díaz-Regañón D, García-Sancho M, Villaescusa A, Sainz Á, Agulla B, Reyes-Prieto M, et al. Characterization of the fecal and mucosa-associated microbiota in dogs with chronic inflammatory enteropathy. Animals. 2023;13(3):326. 10.3390/ani13030326. 36766216 10.3390/ani13030326 PMC9913788

[96] Kiełbik P, Witkowska-Piłaszewicz O. The relationship between canine behavioral disorders and gut microbiome and future therapeutic perspectives. Animals. 2024;14(14):2048. 10.1007/s12275-024-00121-4. 39061510 10.3390/ani14142048 PMC11273744

[97] Yeh YM, Lye XY, Lin HY, Wong JY, Wu CC, Huang CL, et al. Effects of *Lactiplantibacillus plantarum* PS128 on alleviating canine aggression and separation anxiety. Appl Anim Behav Sci. 2022;247:105569. 10.1016/j.applanim.2022.105569.

[98] Patel KV, Hunt AB, Castillo-Fernandez J, Abrams C, King T, Watson P, et al. Impact of acute stress on the canine gut microbiota. Sci Rep. 2024;14:18897. 10.1038/s41598-024-66652-3. 39143116 10.1038/s41598-024-66652-3 PMC11324789

[99] Cannas S, Tonini B, Belà B, Di Prinzio R, Pignataro G, Di Simone D, et al. Effect of a novel nutraceutical supplement (Relaxigen Pet dog) on the fecal microbiome and stress-related behaviors in dogs: a pilot study. J Vet Behav. 2021;42:37–47. 10.1016/j.jveb.2020.09.002.

[100] Hernandez J, Rhimi S, Kriaa A, Mariaule V, Boudaya H, Drut A, et al. Domestic environment and gut microbiota: lessons from pet dogs. Microorganisms. 2022;10(5):949. 10.3390/microorganisms10050949. 35630391 10.3390/microorganisms10050949 PMC9143008

[101] Dergance AE. Investigating the impact of shelter stay on the gut microbiome of dogs. Bachelor's thesis, University of Arizona, Tucson, USA; 2024. http://hdl.handle.net/10150/672849.

[102] Sandri M, Dal Monego S, Conte G, Sgorlon S, Stefanon B. Raw meat based diet influences faecal microbiome and end products of fermentation in healthy dogs. BMC Vet Res. 2017;13:65. 10.1186/s12917-017-0981-z. 28245817 10.1186/s12917-017-0981-z PMC5331737

[103] Hsiao EY, McBride SW, Hsien S, Sharon G, Hyde ER, McCue T, et al. Microbiota modulate behavioral and physiological abnormalities associated with neurodevelopmental disorders. Cell. 2013;155(7):1451–63. 10.1016/j.cell.2013.11.024. 24315484 10.1016/j.cell.2013.11.024 PMC3897394

[104] Czarnecki-Maulden G. Effect of dietary modulation of intestinal microbiota on reproduction and early growth. Theriogenology. 2008;70(3):286–90. 10.1016/j.theriogenology.2008.05.041. 18533245 10.1016/j.theriogenology.2008.05.041

[105] Thomson P, Santibáñez R, Rodríguez-Salas C, Flores-Yañez C, Garrido D. Differences in the composition and predicted functions of the intestinal microbiome of obese and normal weight adult dogs. PeerJ. 2022;10:e12695. 10.7717/peerj.12695. 35190784 10.7717/peerj.12695 PMC8857902

[106] Kang A, Kwak MJ, Lee DJ, Lee JJ, Kim MK, Song M, et al. Dietary supplementation with probiotics promotes weight loss by reshaping the gut microbiome and energy metabolism in obese dogs. Microbiol Spectr. 2024;12(3):e0255223. 10.1128/spectrum.02552-23. 38270436 10.1128/spectrum.02552-23 PMC10913549

[107] Baillon MLA, Marshall-Jones ZV, Butterwick RF. Effects of probiotic *Lactobacillus acidophilus* strain DSM13241 in healthy adult dogs. Am J Vet Res. 2004;65(3):338–43. 10.2460/ajvr.2004.65.338. 15027683 10.2460/ajvr.2004.65.338

[108] Al-Shawi SG, Dang DS, Yousif AY, Al-Younis ZK, Najm TA, Matarneh SK. The potential use of probiotics to improve animal health, efficiency, and meat quality: a review. Agriculture. 2020;10(10):452. 10.3390/agriculture10100452.

[109] Gardiner GE, Metzler-Zebeli BU, Lawlor PG. Impact of intestinal microbiota on growth and feed efficiency in pigs: a review. Microorganisms. 2020;8(12):1886. 10.3390/microorganisms8121886. 33260665 10.3390/microorganisms8121886 PMC7761281

[110] Sanders M, Klaenhammer T. Invited review: the scientific basis of *Lactobacillus acidophilus* NCFM functionality as a probiotic. J Dairy Sci. 2001;84(2):319–31. 10.3168/jds.s0022-0302(01)74481-5. 11233016 10.3168/jds.S0022-0302(01)74481-5

[111] Chun JL, Ji SY, Lee SD, Lee YK, Kim B, Kim KH. Difference of gut microbiota composition based on the body condition scores in dogs. J Anim Sci Technol. 2020;62(2):239. 10.5187/jast.2020.62.2.239. 32292931 10.5187/jast.2020.62.2.239 PMC7142278

[112] Ley RE, Turnbaugh PJ, Klein S, Gordon JI. Microbial ecology: human gut microbes associated with obesity. Nature. 2006;444(7122):1022–3. 10.1038/4441022a. 17183309 10.1038/4441022a

[113] Pedersen R, Andersen AD, Hermann-Bank ML, Stagsted J, Boye M. The effect of high-fat diet on the composition of the gut microbiota in cloned and non-cloned pigs of lean and obese phenotype. Gut Microbes. 2013;4(5):371–81. 10.4161/gmic.26108. 23974297 10.4161/gmic.26108 PMC3839981

[114] Han GG, Lee J-Y, Jin G-D, Park J, Choi YH, Chae BJ, et al. Evaluating the association between body weight and the intestinal microbiota of weaned piglets via 16S rRNA sequencing. Appl Microbiol Biotechnol. 2017;101:5903–11. 10.1007/s00253-017-8304-7. 28523395 10.1007/s00253-017-8304-7

[115] Jiao S, Cao H, Dai Y, Wu J, Lv J, Du R, et al. Effect of high-fat diet and growth stage on the diversity and composition of intestinal microbiota in healthy bovine livestock. J Sci Food Agric. 2017;97(14):5004–13. 10.1002/jsfa.8380. 28417460 10.1002/jsfa.8380

[116] Myer PR. Bovine genome-microbiome interactions: metagenomic frontier for the selection of efficient productivity in cattle systems. mSystems. 2019;4(3):e00103. 10.1128/mSystems.00103-19. 31219789 10.1128/mSystems.00103-19 PMC6533375

[117] Du D, Wang Y, Gao Y, Feng L, Zhang Z, Hu Z. Analysis of differences in the rumen microbiome and metabolic function in prepartum dairy cows with different body condition scores. Anim Microbiome. 2024;6:35. 10.1186/s42523-024-00324-5. 38915057 10.1186/s42523-024-00324-5 PMC11194928

[118] Ma X, Brinker E, Graff EC, Cao W, Gross AL, Johnson AK, et al. Whole-genome shotgun metagenomic sequencing reveals distinct gut microbiome signatures of obese cats. Microbiol Spectr. 2022;10(3): e0083722. 10.1128/spectrum.00837-22. 35467389 10.1128/spectrum.00837-22 PMC9241680

[119] Kim SW, Park KY, Kim B, Kim E, Hyun CK. *Lactobacillus rhamnosus* GG improves insulin sensitivity and reduces adiposity in high-fat diet-fed mice through enhancement of adiponectin production. Biochem Biophys Res Commun. 2013;431(2):258–63. 10.1016/j.bbrc.2012.12.121. 23313485 10.1016/j.bbrc.2012.12.121

[120] Zhang M, Wang C, Wang C, Zhao H, Zhao C, Chen Y, et al. Enhanced AMPK phosphorylation contributes to the beneficial effects of *Lactobacillus rhamnosus* GG supernatant on chronic-alcohol-induced fatty liver disease. J Nutr Biochem. 2015;26(4):337–44. 10.1016/j.jnutbio.2014.10.016. 25622859 10.1016/j.jnutbio.2014.10.016 PMC6679353

[121] Honneffer JB, Minamoto Y, Suchodolski JS. Microbiota alterations in acute and chronic gastrointestinal inflammation of cats and dogs. World J Gastroenterol. 2014;20(44):16489–97. 10.3748/wjg.v20.i44.16489. 25469017 10.3748/wjg.v20.i44.16489 PMC4248192

[122] Minamoto Y, Otoni CC, Steelman SM, Büyükleblebici O, Steiner JM, Jergens AE, et al. Alteration of the fecal microbiota and serum metabolite profiles in dogs with idiopathic inflammatory bowel disease. Gut microbes. 2015;6(1):33–47. 10.1080/19490976.2014.997612. 25531678 10.1080/19490976.2014.997612 PMC4615558

[123] Rhimi S, Kriaa A, Mariaule V, Saidi A, Drut A, Jablaoui A, et al. The nexus of diet, gut microbiota and inflammatory bowel diseases in dogs. Metabolites. 2022;12(12):1176. 10.3390/metabo12121176. 36557214 10.3390/metabo12121176 PMC9782517

[124] Xu H, Huang W, Hou Q, Kwok L-Y, Laga W, Wang Y, et al. Oral administration of compound probiotics improved canine feed intake, weight gain, immunity and intestinal microbiota. Front Immunol. 2019;10:394673. 10.3389/fimmu.2019.00666. 10.3389/fimmu.2019.00666 PMC6454072 31001271

[125] Jaffey JA, Okwumabua O, Graves TK, Al-Nakkash L, Monasky R, Wilson A, et al. Evaluation of *Candida* spp. and other fungi in feces from dogs with naturally occurring diabetes mellitus. Vet Sci. 2022;9(10):567. 10.3390/vetsci9100567. 36288180 10.3390/vetsci9100567 PMC9609726

[126] Kwong TC, Chau ECT, Mak MCH, Choy CT, Chan LT, Pang CK, et al. Characterization of the gut microbiome in healthy dogs and dogs with diabetes mellitus. Animals. 2023;13(15):2479. 10.3390/ani13152479. 37570288 10.3390/ani13152479 PMC10417117

[127] Jergens AE, Guard BC, Redfern A, Rossi G, Mochel JP, Pilla R, et al. Microbiota-related changes in unconjugated fecal bile acids are associated with naturally occurring, insulin-dependent diabetes mellitus in dogs. Front Vet Sci. 2019;6:199. 10.3389/fvets.2019.00199. 31316997 10.3389/fvets.2019.00199 PMC6610424

[128] Laia NL, Barko PC, Sullivan DR, McMichael MA, Williams DA, Reinhart JM. Longitudinal analysis of the rectal microbiome in dogs with diabetes mellitus after initiation of insulin therapy. PLoS ONE. 2022;17(9):e0273792. 10.1371/journal.pone.0273792. 36067170 10.1371/journal.pone.0273792 PMC9447884

[129] Giuffrè M, Campigotto M, Campisciano G, Comar M, Crocè LS. A story of liver and gut microbes: How does the intestinal flora affect liver disease? A review of the literature. Am J Physiol Gastrointest Liver Physiol. 2020;318(5):G889–906. 10.1152/ajpgi.00161.2019. 32146836 10.1152/ajpgi.00161.2019

[130] Cerquetella M, Mangiaterra S, Pinnella F, Rossi G, Marchegiani A, Gavazza A, et al. Fecal proteome profile in dogs suffering from different hepatobiliary disorders and comparison with controls. Animals. 2023;13(14):2343. 10.3390/ani13142343. 37508119 10.3390/ani13142343 PMC10376375

[131] Habermaass V, Biolatti C, Bartoli F, Gori E, Bruni N, Olivero D, et al. Effects of synbiotic administration on gut microbiome and fecal bile acids in dogs with chronic hepatobiliary disease: a randomized case–control study. Vet Sci. 2024;11(8):364. 10.3390/vetsci11080364. 39195817 10.3390/vetsci11080364 PMC11360150

[132] Rostaher A, Morsy Y, Favrot C, Unterer S, Schnyder M, Scharl M, et al. Comparison of the gut microbiome between atopic and healthy dogs—preliminary data. Animals. 2022;12(18):2377. 10.3390/ani12182377. 36139237 10.3390/ani12182377 PMC9495170

[133] De Pessemier B, Grine L, Debaere M, Maes A, Paetzold B, Callewaert C. Gut–skin axis: current knowledge of the interrelationship between microbial dysbiosis and skin conditions. Microorganisms. 2021;9(2):353. 10.3390/microorganisms9020353. 33670115 10.3390/microorganisms9020353 PMC7916842

[134] Mahmud MR, Akter S, Tamanna SK, Mazumder L, Esti IZ, Banerjee S, et al. Impact of gut microbiome on skin health: gut-skin axis observed through the lenses of therapeutics and skin diseases. Gut microbes. 2022;14(1):2096995. 10.1080/19490976.2022.2096995. 35866234 10.1080/19490976.2022.2096995 PMC9311318

[135] Craig JM. Atopic dermatitis and the intestinal microbiota in humans and dogs. Vet Med Sci. 2016;2(2):95–105. 10.1002/vms3.24. 29067183 10.1002/vms3.24 PMC5645856

[136] Thomsen M, Künstner A, Wohlers I, Olbrich M, Lenfers T, Osumi T, et al. A comprehensive analysis of gut and skin microbiota in canine atopic dermatitis in Shiba Inu dogs. Microbiome. 2023;11:232. 10.1186/s40168-023-01671-2. 37864204 10.1186/s40168-023-01671-2 PMC10590023

[137] Schmidt T, Meller S, Meyerhoff N, Twele F, Zanghi B, Volk HA. A six-month prospective, randomised, double-blinded, placebo-controlled, crossover, dietary trial design to investigate the potential of psychobiotics on seizure semiology and comorbidities in canine epilepsy: study protocol. BMC Vet Res. 2023;19(1):57. 10.1186/s12917-023-03609-0. 36864510 10.1186/s12917-023-03609-0 PMC9983181

[138] Bravo JA, Forsythe P, Chew MV, Escaravage E, Savignac HM, Dinan TG, et al. Ingestion of *Lactobacillus* strain regulates emotional behavior and central GABA receptor expression in a mouse via the vagus nerve. Proc Natl Acad Sci U S A. 2011;108(38):16050–5. 10.1073/pnas.1102999108. 21876150 10.1073/pnas.1102999108 PMC3179073

[139] Beerda B, Schilder MB, van Hooff JA, de Vries HW. Manifestations of chronic and acute stress in dogs. Appl Anim Behav Sci. 1997;52(3–4):307–19. 10.1016/s0168-1591(96)01131-8.

[140] Slykerman R, Hood F, Wickens K, Thompson J, Barthow C, Murphy R, et al. Effect of *Lactobacillus rhamnosus* HN001 in pregnancy on postpartum symptoms of depression and anxiety: a randomised double-blind placebo-controlled trial. EBioMedicine. 2017;24:159–65. 10.1016/j.ebiom.2017.09.013. 28943228 10.1016/j.ebiom.2017.09.013 PMC5652021

[141] Işık M, Köse F, Özbayer C, Budak Ö, Kaya RK, Erdoğan DG, et al. Promising antidepressant potential: the role of *Lactobacillus rhamnosus* GG in mental health and stress response. Probiotics Antimicro Pro. 2025:1–31. 10.1007/s12602-025-10470-0. 10.1007/s12602-025-10470-0 PMC12634810 39962033

[142] Yang Q, Wu Z. Gut probiotics and health of dogs and cats: benefits, applications, and underlying mechanisms. Microorganisms. 2023;11(10):2452. 10.3390/microorganisms11102452. 37894110 10.3390/microorganisms11102452 PMC10609632

[143] Doo H, Kwak J, Keum GB, Ryu S, Choi Y, Kang J, et al. Lactic acid bacteria in Asian fermented foods and their beneficial roles in human health. Food Sci Biotechnol. 2024:1–13. 10.1007/s10068-024-01634-9. 10.1007/s10068-024-01634-9 PMC11315863 39130665

[144] Kim S, Cho J, Keum GB, Kwak J, Doo H, Choi Y, et al. Investigation of the impact of multi-strain probiotics containing *Saccharomyces cerevisiae* on porcine production. J Anim Sci Technol. 2024;66(5):876. 10.5187/jast.2024.e79. 39398307 10.5187/jast.2024.e79 PMC11466735

[145] Marsella R, Santoro D, Ahrens K. Early exposure to probiotics in a canine model of atopic dermatitis has long-term clinical and immunological effects. Vet Immunol Immunopathol. 2012;146(2):185–9. 10.1016/j.vetimm.2012.02.013. 22436376 10.1016/j.vetimm.2012.02.013

[146] Holscher HD. Dietary fiber and prebiotics and the gastrointestinal microbiota. Gut Microbes. 2017;8(2):172–84. 10.1080/19490976.2017.1290756. 28165863 10.1080/19490976.2017.1290756 PMC5390821

[147] Delgado GTC, Tamashiro WMdSC. Role of prebiotics in regulation of microbiota and prevention of obesity. Food Res Int. 2018;113:183–8. 10.1016/j.foodres.2018.07.013. 30195512 10.1016/j.foodres.2018.07.013

[148] Tal S, Tikhonov E, Aroch I, Hefetz L, Turjeman S, Koren O, et al. Developmental intestinal microbiome alterations in canine fading puppy syndrome: a prospective observational study. npj Biofilms Microbiomes. 2021;7(1):52. 10.1038/s41522-021-00222-7. 34162880 10.1038/s41522-021-00222-7 PMC8222291

[149] Connelly S, Fanelli B, Hasan NA, Colwell RR, Kaleko M. Syn-007, an orally administered beta-lactamase enzyme, protects the gut microbiome from oral amoxicillin/clavulanate without adversely affecting antibiotic systemic absorption in dogs. Microorganisms. 2020;8(2):152. 10.3390/microorganisms8020152. 10.3390/microorganisms8020152 PMC7074739 31979034

[150] Tuniyazi M, Hu X, Fu Y, Zhang N. Canine fecal microbiota transplantation: current application and possible mechanisms. Vet sci. 2022;9(8):396. 10.3390/vetsci9080396. 36006314 10.3390/vetsci9080396 PMC9413255

